# Concordant Regulation of Translation and mRNA Abundance for Hundreds of Targets of a Human microRNA

**DOI:** 10.1371/journal.pbio.1000238

**Published:** 2009-11-10

**Authors:** David G. Hendrickson, Daniel J. Hogan, Heather L. McCullough, Jason W. Myers, Daniel Herschlag, James E. Ferrell, Patrick O. Brown

**Affiliations:** 1 Department of Chemical and Systems Biology, Stanford University School of Medicine, Stanford, California, United States of America; 2 Department of Biochemistry, Stanford University School of Medicine, Stanford, California, United States of America; 3 Howard Hughes Medical Institute, Stanford University School of Medicine, Stanford, California, United States of America; 4 Department of Genetics, Stanford University School of Medicine, Stanford, California, United States of America; University of Massachusetts Medical School, United States of America

## Abstract

MicroRNAs (miRNAs) regulate gene expression posttranscriptionally by interfering with a target mRNA's translation, stability, or both. We sought to dissect the respective contributions of translational inhibition and mRNA decay to microRNA regulation. We identified direct targets of a specific miRNA, miR-124, by virtue of their association with Argonaute proteins, core components of miRNA effector complexes, in response to miR-124 transfection in human tissue culture cells. In parallel, we assessed mRNA levels and obtained translation profiles using a novel global approach to analyze polysomes separated on sucrose gradients. Analysis of translation profiles for ∼8,000 genes in these proliferative human cells revealed that basic features of translation are similar to those previously observed in rapidly growing *Saccharomyces cerevisiae*. For ∼600 mRNAs specifically recruited to Argonaute proteins by miR-124, we found reductions in both the mRNA abundance and inferred translation rate spanning a large dynamic range. The changes in mRNA levels of these miR-124 targets were larger than the changes in translation, with average decreases of 35% and 12%, respectively. Further, there was no identifiable subgroup of mRNA targets for which the translational response was dominant. Both ribosome occupancy (the fraction of a given gene's transcripts associated with ribosomes) and ribosome density (the average number of ribosomes bound per unit length of coding sequence) were selectively reduced for hundreds of miR-124 targets by the presence of miR-124. Changes in protein abundance inferred from the observed changes in mRNA abundance and translation profiles closely matched changes directly determined by Western analysis for 11 of 12 proteins, suggesting that our assays captured most of miR-124–mediated regulation. These results suggest that miRNAs inhibit translation initiation or stimulate ribosome drop-off preferentially near the start site and are not consistent with inhibition of polypeptide elongation, or nascent polypeptide degradation contributing significantly to miRNA-mediated regulation in proliferating HEK293T cells. The observation of concordant changes in mRNA abundance and translational rate for hundreds of miR-124 targets is consistent with a functional link between these two regulatory outcomes of miRNA targeting, and the well-documented interrelationship between translation and mRNA decay.

## Abstract 2

A specific microRNA reduces the synthesis of hundreds of proteins via concordant effects on the abundance and translation of the mRNAs that encode them.

## Author Summary

The human genome contains directions to regulate the timing and magnitude of expression of its thousands of genes. MicroRNAs are important regulatory RNAs that tune the expression levels of tens to hundreds of specific genes by pairing to complimentary stretches in the messenger RNAs from these genes, thereby reducing their stability and their translation into protein. Although the importance of microRNAs is appreciated, little is known about the relative contributions of degradation or repression of translation of the cognate mRNAs to the overall effects on protein synthesis, or the links between these two regulatory mechanisms. We devised a simple, economical method to systematically measure mRNA translation profiles, then applied this method, in combination with gene expression analysis, to measure the effects of the human microRNA miR-124 on the abundance and apparent translation rate of its mRNA targets. We found that for the ∼600 mRNA targets of miR-124 that were identified by their association with microRNA effector complexes, around three quarters of the reduction in estimated protein synthesis was explained by changes in mRNA abundance. Although the apparent changes in translation efficiencies of the targeted mRNAs were smaller in magnitude, they were highly correlated with changes in the abundance of those RNAs, suggesting a functional link between microRNA-mediated repression of translation and mRNA decay.

## Introduction

MicroRNAs (miRNAs) are small noncoding RNAs whose complementary pairing to target mRNAs potentially regulates expression of more than 60% of genes in many and perhaps all metazoans [1]–[6]. Destabilization of mRNA and translational repression have been suggested as the mechanisms of action for miRNAs [1],[3],[7]–[15], and recent work directly measuring endogenous protein levels in response to altered miRNA expression levels found that specific miRNAs modestly inhibit the production of hundreds of proteins [16],[17].

The importance and functional range of miRNAs are evidenced by the diverse and often dramatic phenotypic consequences when miRNAs are mutated or misexpressed, leading to aberrant development or disease [7],[18]–[24]. Although regulation by miRNAs is an integral component of the global gene expression program, there is currently no consensus on either the mechanism by which they decrease the levels of the targeted proteins or even the steps in gene expression regulated by miRNAs [3],[25]–[29].

The proposal that miRNAs decrease protein levels without affecting mRNA stability arose from the observation that the miRNA *lin-4* down-regulates *lin-14* expression in the absence of noticeable changes in *lin-14* mRNA abundance in *Caenorhabditis elegans* [7],[30]–[33]. Subsequent studies in mammalian cell culture provided further support for this model [34]–[37]. Several studies have found that repressed mRNAs as well as protein components of the miRNA regulatory system accumulate in P-bodies, suggesting that repressed mRNAs may be sequestered away from the translation pool [38]–[44]. Other evidence points to deadenylation of miRNA-targeted mRNAs, an effect that can inhibit translation [14],[45]–[53]. Some studies have argued that initiation of translation is blocked at either an early, cap-dependent stage or later during AUG recognition or 60S joining [10],[44],[52],[54]–[58]. Others have argued that a postinitiation step is targeted, resulting in either slowed elongation, ribosome drop-off, or nascent polypeptide degradation [7],[59]–[62].

One factor contributing to the lack of a consensus model for miRNA function is the evidence that miRNA targeting of an mRNA significantly reduces message levels (despite previous reports to the contrary) [9],[11],[12],[14],[52],[63],[64]. Indeed, very recent studies from Baek et al. and Selbach et al. found that the changes in mRNA abundance are not only correlated with the repression of many targets, but also can account for most of the observed reduction in protein expression [16],[17]. mRNA targets of the same miRNA can either be translationally repressed with little change in mRNA abundance, translationally repressed and have concordant changes in mRNA abundance, or have little translation repression with large changes in mRNA abundance [52],[65],[66]. That miRNAs can affect both protein production and abundance of their mRNA targets raises the question of to what extent these outcomes of miRNA regulation are mediated by a common mechanism or by competing or complementary processes. The regulatory consequence of a particular miRNA–mRNA interaction might be influenced by miRNA-independent factors such as cellular context or by additional information encoded by the target mRNA, e.g., presence of binding sites for other RNA-binding proteins and miRNAs, secondary structure around miRNA binding sites, or the intrinsic decay rate of the mRNA [25],[51],[67],[68].

Experiment-specific effects of in vitro translation assays, reporter constructs, or procedural differences that alter properties of gene expression could account for some of the wide variation in the apparent mechanisms by which miRNAs alter expression [25],[27]. To date, most studies on translational regulation by miRNAs have used reporter assays. Although assays that rely on engineered reporter transcripts are powerful, assay-specific anomalies are a concern; artificial mRNAs may lack key pieces of regulatory information, overexpression of reporter mRNAs could mask subtle regulatory functions, and DNA transfection can lead to indirect effects on cell physiology [26]. Indeed, recent reports have found that differences in experimental setup, such as the method of transfection, type of 5′-cap, or the promoter sequence of the DNA reporter construct can drastically alter the degree or even the apparent mode of regulation by miRNAs [59],[69]. In addition, some models have been based on studies in which only one or a few targets were studied, which introduces the possibility of generalizing the behavior of a single miRNA–mRNA interaction that may not represent the dominant biological mechanism.

Two recent studies avoided many of these caveats by overexpressing, inhibiting, or deleting specific miRNAs and systematically measuring changes in endogenous mRNA and protein levels using DNA microarrays and stable isotope labeling with amino acids in cell culture (SILAC), respectively [16],[17]. Both studies found mostly concordant changes in mRNA levels and protein levels, with changes in mRNA levels accounting for much, but not all, of the changes in protein abundance. With data for hundreds of endogenous targets, these studies were the first to provide genome-wide evidence that mRNA degradation accounts for much of the reduction in protein levels. And whereas these results suggest that translation inhibition accounts for some of the observed changes in protein abundance of miRNA targets, they do not provide direct evidence of this, nor do they provide insight into which steps in translation are regulated, the extent this regulation contributes to reduced gene expression of specific mRNAs, or its possible links to mRNA decay.

To investigate how miRNAs regulate gene expression, we systematically identified direct targets of the miRNA miR-124 by measuring the recruitment of target mRNAs to Argonaute (Ago) proteins, the core components of the miRNA effector complex, as previously described [70]–[72]. We then measured, in parallel, mRNA abundance and two indicators of translation rate, ribosome occupancy and ribosome density, for more than 8,000 genes, using DNA microarrays and a novel polysome encoding scheme. This strategy allowed us to directly investigate the behavior of miRNA–mRNA target pairs with respect to both mRNA fate and translation, on a genomic scale.

## Results

### Systematic Identification of mRNAs Recruited to Argonautes by miR-124

To study the effects of miR-124 on expression of mRNA targets, we first had to identify those targets. Recruitment to Ago complexes in response to the expression of a particular miRNA appears to be the most reliable criterion for target identification [70]. To this end, we lysed human embryonic kidney (HEK) 293T cells transfected with miR-124 and isolated Ago-associated RNA by immunopurification (IP) using a monoclonal antibody that recognizes all four human Ago paralogs [73]. We measured mRNA enrichment in Ago IPs by comparative DNA microarray hybridization of samples prepared from immunupurified RNA and total RNA from cell extracts. Three replicates of Ago and control IPs were performed from both miR-124 and mock-transfected cells (Datasets S1 and S5).

To examine the enrichment profiles of the IPs, we first clustered the microarray results by their similarity and visualized the results as a heatmap, with the degree of enrichment of each RNA shown on a green (least enriched) to red (most enriched) scale (Figure S1). The Ago IP enrichment profiles were reproducible as evidenced by an average Pearson correlation coefficient between mRNA enrichment profiles of Ago IPs in mock-transfected cells and miR-124–transfected cells of 0.90 and 0.94, respectively.

Thousands of mRNAs were reproducibly enriched in the Ago IPs from mock-transfected cells (Figures S1 and S2, and Text S1). We found that the presence of sequence matches to two highly expressed microRNA families, miR-17-5p/20/92/106/591.d and miR-19a/b, in the 3′-untranslated regions (UTRs) of mRNAs significantly correlated with Ago IP enrichment (Text S2), suggesting that association with Ago is in large part a reflection of the relative occupancy of each mRNA with the suite of miRNAs endogenously expressed in HEK293T cells. High-confidence Ago-associated mRNAs (at least 4-fold enriched over the mean, 1,363 mRNAs) disproportionately encode regulatory proteins (409, *p* = 0.001), with roles including “transcription factor activity” (95, *p* = 0.01), “signal transduction” (230, *p* = 0.02), and “gene silencing by RNA” (7, *p* = 0.02).

**Figure 1:**
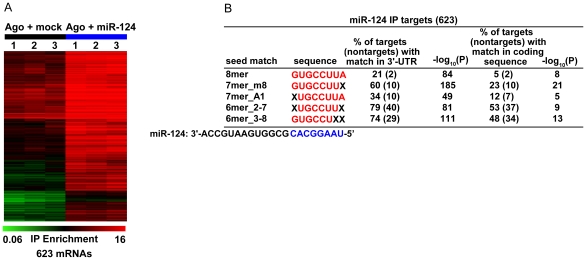
miR-124 recruits hundreds of specific mRNAs to Argonautes. (A) Supervised hierarchical clustering of the enrichment profiles of putative miR-124 Ago IP targets (1% local FDR) in Ago IPs from miR-124–transfected cells (blue) and mock-transfected cells (black). Rows correspond to 789 sequences (representing 623 genomic loci with a RefSeq sequence), and columns represent individual experiments. The color scale encompasses a range from 0.06- to 16-fold relative to global mean IP enrichment for each mRNA (−4 to +4 logs on log base 2 scale ). (B) Enrichment of seed matches to miR-124 in the 3′-UTRs and coding sequences of miR-124 Ago IP targets (1% local FDR). The significance of enrichment of seed matches in Ago IP targets was measured using the hypergeometric distribution function.

To identify RNAs specifically recruited to Agos by miR-124, we compared the mRNA enrichment profiles of Ago IPs from miR-124–transfected cells to Ago IPs from mock-transfected cells using the significance analysis of microarrays (SAM) modified two-sample unpaired *t*-test (Datasets S1 and S5). At a stringent 1% local false-discovery rate (FDR) threshold, we identified 623 distinct mRNAs significantly enriched in Ago IPs from lysates of miR-124–transfected cells compared to Ago IPs from mock-transfected cells (Figure 1A).

Previous work established that the 5′-end of the miRNA, the “seed region,” is particularly important for interactions with mRNA targets [4],[11],[37],[74]–[77]. In most cases, there is a 6–8 bp stretch of perfect complementarity between the seed region of the miRNA and a “seed match” sequence in the 3′-UTR of the mRNA [4],[11],[37],[74]–[77]. We reasoned that if the mRNAs specifically recruited to Agos by miR-124 transfection were physically associated with miR-124, seed match sequences would be significantly enriched in miR-124–specific IP targets compared to nontargets. Indeed, we found strong enrichment of 6–8 base seed matches to miR-124 in the 3′-UTRs of miR-124 Ago IP targets (Figure 1B). We also found enrichment within the coding sequences of miR-124 Ago IP targets, as previously reported (Figure 1B) [11],[16],[17],[70],[71],[78],[79]. For instance, 60% of miR-124 Ago IP targets contain a perfect match to positions 2–8 of miR-124 (called 7mer-m8) in their 3′-UTRs, compared to 10% of nontargets (*p*<10^−185^, hypergeometric distribution), and 23% of miR-124 Ago IP targets contain a perfect match to positions 2–8 of miR-124 in their coding sequence, compared to 10% of nontargets (*p*<10^−21^). After removing mRNAs with 7mer seed matches in their 3′-UTRs, the remaining miR-124 IP targets were still significantly, albeit weakly, enriched for 3′-UTR 6mer matches to miR-124 (6mer 2–7, *p* = 0.008, 6mer 3–8, *p*<10^−5^). These data argue that most miR-124 Ago IP targets were recruited to Agos by direct association with miR-124, via seed matches in their 3′-UTRs or coding sequences.

### Systematic Measurement of mRNA Translation Profiles

The standard approach to assess translation in vivo has been the analysis of “polysome profiles.” After treatment with cycloheximide to trap elongating ribosomes, mRNAs with no associated ribosomes and those with varying numbers of ribosomes bound can be separated by velocity sedimentation through a sucrose gradient. The polysome profile of a gene's mRNA provides information on two key parameters in translation: (1) the fraction of the mRNA species bound by at least one ribosome, and presumably undergoing translation, referred to as “ribosome occupancy,” and (2) the average number of ribosomes bound per 100 bases of coding sequence to mRNAs that have at least one bound ribosome, referred to as the “ribosome density.”

We previously developed a method to systematically measure ribosome occupancy and ribosome density by measuring the relative amount of each gene's mRNA in each fraction of a polysome profile using DNA microarray hybridization [80].

**Figure 2:**
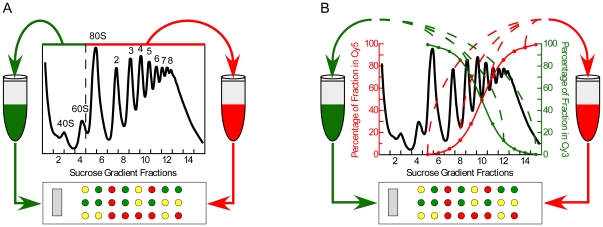
Systematic translation profiling by microarray analysis. (A) Schematic of the procedure used to systematically measure each gene's mRNA ribosome occupancy (see Results and Materials and Methods for details). (B) Schematic of the “gradient encoding” method to measure the average number of ribosomes bound to each gene's mRNAs (see Results and Materials and Methods for details).

We have since developed and implemented a more streamlined approach that uses one DNA microarray hybridization to measure ribosome occupancy and only a single additional microarray hybridization to measure ribosome density (Figure 2 and Figure S3). We measured ribosome occupancy by first pooling ribosome bound fractions and unbound fractions and adding exogenous doping control RNAs to each (Figure 2A). Poly(A) RNA from bound and unbound pools was isolated, amplified, coupled to Cy5 and Cy3 dyes, respectively, and comparatively hybridized to DNA microarrays. The ribosome occupancy for each gene's mRNA was obtained after scaling the microarray data using the doping controls (see Materials and Methods for details).

We determined the ribosome density for each gene's mRNA by a “gradient encoding” strategy in which a graded ratio of each fraction from the ribosome bound fractions was split into a “heavy” and a “light” pool, respectively. For instance, 99% of the first fraction (∼one ribosome bound) was added to the light pool and 1% was added to the heavy pool. Then, 98% of the second fraction (1.5–2 ribosomes bound) was added to the light pool and 2% was added to the heavy pool, and so on, such that the light pool was enriched for mRNAs associated with fewer ribosomes, and the heavy pool was enriched for mRNAs associated with a greater number of ribosomes (Figure 2B). The RNA in each pool was amplified, labeled with Cy5 or Cy3, mixed, and comparatively hybridized to DNA microarrays. Thus, the Cy5/Cy3 ratio measured at each element on the array is a monotonic function of the mass-weighted average sedimentation coefficient of the corresponding mRNA, which is primarily determined by the number of ribosomes bound to it. The validity of this approach is supported by the very strong concordance between ribosome density measurements in yeast obtained with the gradient-encoding method and our previously published ribosome density measurements obtained using the traditional approach of analyzing each fraction on separate DNA microarrays (Pearson *r* = 0.95) (unpublished data) [80]. Further details of the methodology, as well as control experiments and additional analyses, will be described elsewhere.

To measure the effects of miR-124 on translation, we performed translation profiling on cell extracts generated from the same miR-124–transfected, or mock-transfected cell cultures that were used for Ago IPs and mRNA expression profiling (see below). We obtained high-quality ribosome occupancy and ribosome density measurements on 16,140 sequences (representing 10,455 genes) from three independent mock-transfected cultures and two miR-124–transfected cultures (Datasets S2, S3, and S5). There was a strong concordance between replicate experiments for both the ribosome occupancy and ribosome number/density measurements, both in terms of the correlation of the gene-specific measurements (Pearson correlation for ribosome occupancy = 0.85–0.89, ribosome number = 0.91–0.97) and the means (mean ribosome occupancy = 0.83–0.87, mean ribosome number = 5.6–6.1 per mRNA), which were derived independently for each experiment based on the exogenous doping controls.

**Figure 3:**
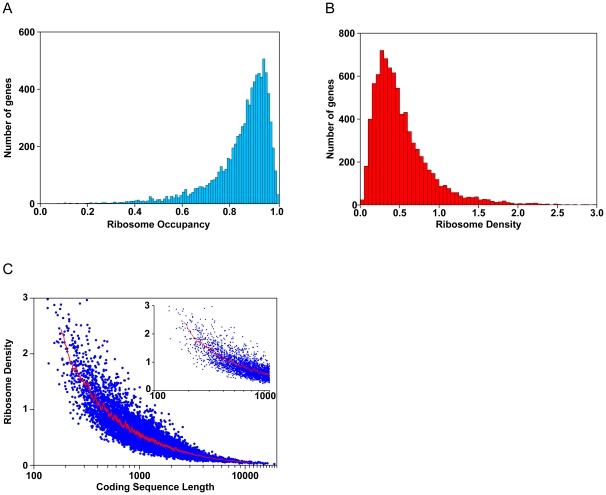
Analysis of ribosome occupancy and ribosome density in HEK293T cells. (A) The number of genes as a function of ribosome occupancy. The average ribosome occupancy is 85%. (B) The number of genes as a function of ribosome density. On average, there is one ribosome per 189 nucleotides. (C) Ribosome density as a function of a gene's coding sequence length. Each gene is indicated by a blue circle. The red line indicates the moving average density value (window of 50). The inset shows only genes with coding sequences that are shorter than 1,000 nucleotides.

The measurements from mock-transfected cells provide some general insights into the translation regulatory program in proliferating human cells. Here, we focus on 8,385 genes that correspond to a RefSeq mRNA for which we obtained high-quality measurements in both Ago IP and mRNA expression DNA microarray experiments. The average ribosome occupancy for the mRNAs from these 8,385 genes was 85% (25th and 75th quartiles = 0.81 and 0.94, respectively) (Figure 3A) suggesting, that for most genes, most polyadenylated mRNAs are associated with ribosomes under these growth conditions and that there are not abundant pools of polyadenylated mRNAs in an untranslated “compartment.” For more than 97% of the genes analyzed, a majority of the transcripts were associated with ribosomes; mRNA transcripts of 3% of these genes (224) were predominantly unassociated with ribosomes (ribosome occupancy <50%). The reason for the relative exclusion of this small set of mRNAs from the highly translated pool remains to be determined: possibilities include sequestration from the translation machinery or a relatively short half-life that results in these mRNAs spending a correspondingly small fraction of their lives in the translated pool. We searched for common biological themes among these non–ribosome-associated mRNAs using gene ontology (GO) term analysis, and found that an unexpectedly large fraction of these mRNAs encode proteins involved in “regulation of transcription” (64, *p*<10^−7^). On the flip side, there were 342 genes whose mRNAs were almost completely (98% or greater) associated with ribosomes. Many of these mRNAs encoded proteins involved in metabolism and gene expression, including “oxidative phosphorylation” (21, *p*<10^−10^), “nuclear mRNA splicing” (23, *p*<10^−5^), “proteasome complex” (11, *p* = 0.0003), and “glycolysis” (10, *p* = 0.0002). mRNAs with low ribosome occupancy (less than 50%) were significantly less abundant than mRNAs with high ribosome occupancy (greater than 98%) (Kolmogorov-Smirnov test, *p*<10^−15^), consistent with the hypothesis that a lower rate of decay, and hence a greater fraction of the lifespan spent in the translated pool, contributes to ribosome occupancy.

The average ribosome density for the 8,385 genes with technically high-quality data across this set of experiments was 0.53 ribosomes per 100 nucleotides (nts) (25th and 75th quartiles = 0.27 and 0.67, respectively), which corresponds to one ribosome per 189 nts (Figure 3B). Given that ribosomes are believed to span ∼30 nts of the mRNA, the average ribosome density would be approximately one sixth of the maximal packing density [81]. This spacing suggests that translation initiation is rate limiting for most mRNAs.

We previously observed a strong negative correlation between an mRNA's ribosome density and its coding sequence length in yeast cells rapidly growing in rich medium [80]. Subsequent experiments suggested that this relationship is due to either a strong inverse correlation between initiation rate and coding sequence length [82], or a decrease in ribosome density as a function of position along the mRNA [83]. We found the same inverse relationship between the size of a coding sequence and ribosome density in proliferating mammalian cells (Spearman *r* = −0.90) (Figure 3C). Sucrose gradient sedimentation did not clearly resolve polysomes containing more than seven ribosomes, so it is possible that our method underestimates the number of ribosome bound to mRNAs with long coding sequences, which could, in principle, lead to a spurious negative correlation between coding sequence length and ribosome density. However, the inverse relationship between coding sequence length and ribosome density is still readily evident when only mRNAs with coding sequences less than 1,000 nts are considered (*r* = −0.73), strongly supporting the validity of this relationship.

These broad similarities between translational programs in proliferating HEK293T cells and proliferating *S. cerevisiae* grown in rich medium, suggest that the overall organization of the program, and perhaps some of the fundamental mechanisms underlying the regulation of translation, may be similar in rapidly growing yeast and human cells [80].

### mRNA Recruitment to Argonautes by miR-124 Leads to Modest Decreases in Abundance and Translation Rate

To measure the effects of miR-124 on mRNA expression levels, we profiled mRNA expression in the same cell cultures that we used for the Ago IPs and translation profiling. We obtained high-quality measurements for 15,301 genes from three independent mock-transfected cultures and three independent miR-124–transfected cultures (Datasets S4 and S5). There was strong concordance between replicate experiments (Pearson *r* = 0.95–0.97).

**Figure 4:**
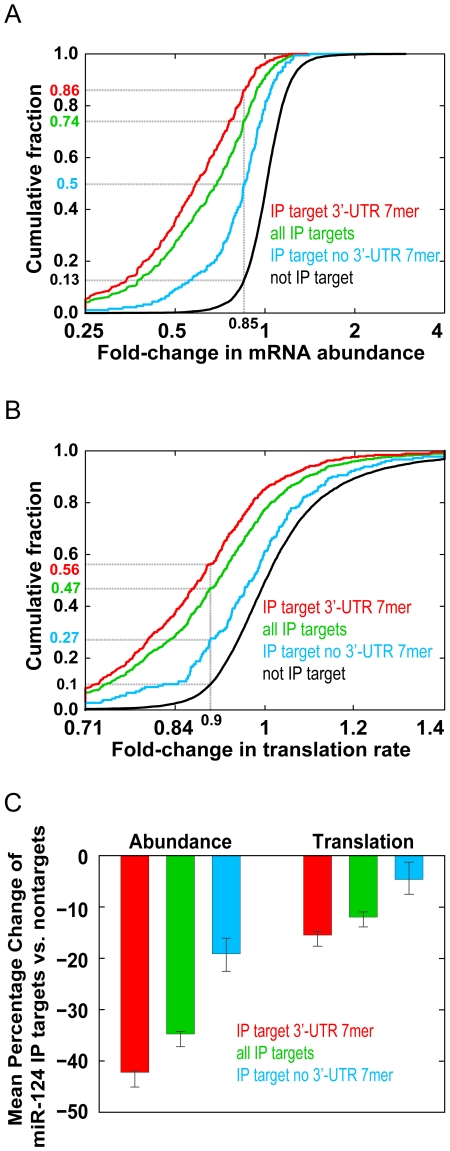
miR-124 negatively regulates the abundance and translation of mRNA targets. (A) Cumulative distribution of the change in mRNA levels following transfection with miR-124 compared to mock. This analysis compares miR-124 Ago IP targets (1% local FDR) (560, green), IP targets with at least one 3′-UTR 7mer seed match (379, red), IP targets that lacked a 3′-UTR 7 mer seed match (181, blue), and nontargets (7,825, black). mRNA levels of miR-124 Ago IP targets were more likely than nontargets to decrease following transfection with miR-124 (*p*<10^−173^). (B) Cumulative distribution of the change in the estimated translation rate following transfection with miR-124 compared to mock. This analysis compares miR-124 Ago IP targets (1% local FDR) (green), IP targets with at least one 3′-UTR 7 mer seed match (red), IP targets that lacked a 3′-UTR 7mer seed match (blue), and nontargets (black). Translation rates of miR-124 Ago IP targets were more likely than nontargets to decrease following transfection with miR-124 (*p*<10^−61^). (C) Bar plot showing the average change in mRNA abundance (left) and translation rate (right) following transfection with miR-124 of all miR-124 Ago IP targets (green), IP targets with at least one 3′-UTR 7mer seed match (red), IP targets that lacked a 3′-UTR 7mer seed match (blue). The average change in mRNA abundance and translation of targets was calculated by subtracting the average change of nontargets for the mRNA abundance and translation rate measurements following transfection with miR-124. The error bars represent 95% confidence intervals in the mean difference estimated by bootstrap analysis.

To study the effects of miR-124 on the expression of its mRNA targets, we first compared the changes in mRNA abundance of Ago IP targets of miR-124 (560 mRNAs; 1% local FDR) and nontargets (7,825 mRNAs) between cells transfected with miR-124 and cells that were mock transfected. Samples were taken 12 h after the respective treatments. We plotted the cumulative distributions of miR-124–dependent Ago IP targets (Figure 4A, green curve) and nontarget mRNAs (Figure 4A, black curve) as a function of the differences in their mRNA abundance between miR-124 and mock-transfected cells. miR-124 target mRNAs were much more likely to decrease in abundance after miR-124 transfection than nontargets (*p*<10^−173^, one-sided Kolmogorov-Smirnov test). For example, 74% of miR-124 IP targets decreased at least 15% at the mRNA level, compared to 13% of nontargets. The average abundance of miR-124 Ago IP targets decreased by 35% compared to nontargets (Figure 4C, green bar on the left). The results are consistent with miRNAs having significant, but modest effects on the mRNA levels of most of their endogenous mRNA targets.

Previous work has established that perfect seed matches to the miRNA in 3′-UTRs are important to elicit effects on mRNA abundance [4],[11],[37],[74]–[77]. To test the importance of 3′-UTR seed matches on the expression of miR-124 targets, we plotted the cumulative distributions of miR-124 IP targets with at least one 7mer 3′-UTR seed match (379, Figure 4A, red curve) and miR-124 IP targets that lacked a 7mer 3′-UTR seed match (181, Figure 4A, blue curve). We found that mRNA targets with 7mer 3′-UTR seed matches were more likely than targets that lacked a 7mer 3′-UTR seed match to decrease in abundance in the presence of miR-124 (90% of miR-124 IP targets with a 3′-UTR seed match decreased at least 15%, compared to 49% of targets that lacked a 7mer 3′-UTR seed match). On average, IP target mRNAs with 7mer 3′-UTR seed matches decreased 40%, whereas IP targets that did not have a 7mer seed match in their 3′-UTR decreased 17%, compared to nontargets (Figure 4C, left). These results underscore the importance of 3′-UTR seed matches for regulation at the mRNA level, but also demonstrate that a large fraction of miR-124 IP targets that lack 7mer seed matches to miR-124 in their 3′-UTR are nevertheless regulated at the mRNA level by miR-124.

To study the effects of miR-124 on translation of targeted mRNAs, we estimated the change in the translation rates between miR-124-transfected and mock-transfected cells (*Tr*) for each mRNA as:
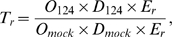
(1)where multiplying *O*, the fraction of the mRNA that is ribosome-bound (ribosome occupancy), by *D*, the average number of ribosomes per 100 nts for bound mRNAs (ribosome density) provides the weighted ribosome density for each mRNA; *Er* is an unmeasured value for the elongation rate of any given mRNA and was assumed not to change (discussed further below). Values *Tr* obtained from miR-124 transfected cells were divided by those from mock-transfected cells to estimate the change. We plotted the cumulative distribution of *Tr* for miR-124 Ago IP targets and nontargets (Figure 4B). miR-124 targets (Figure 4B, green curve) were much more likely to decrease in translation rate than nontarget mRNAs (Figure 4B, black curve) (*p*<10^−62^, one-sided Kolmogorov-Smirnov test). The apparent translation rate of 47% of miR-124 Ago IP targets, but only 10% of nontargets, decreased by at least 10%. In line with what we observed for changes in mRNA abundance, miR-124 IP targets with at least one 7mer seed match in their 3′-UTR were more likely to decrease in translation rate than miR-124 IP targets that lacked a 7mer 3′-UTR seed match (56% percent of miR-124 IP targets with a 7mer 3′-UTR seed match decreased at least 10% in translation versus 27% of IP targets that lacked a 7mer 3′-UTR seed match). The overall effects on translation, while highly significant, were very modest; on average, the estimated translation rates of miR-124 Ago IP targets decreased by 12% relative to nontargets (15% for miR-124 IP targets with a 7mer 3′-UTR seed match and 5% for miR-124 IP targets without a 7mer 3′-UTR seed match) (Figure 4C, right). These results show that miR-124 has modest effects on the abundance, translation rate, or both for most its targets.

In some cases, mRNAs that are translationally repressed are deadenylated and stored, rather than degraded [84]–[86]. All of our measurements were of mRNAs amplified based on their poly(A) tails. Therefore, it was possible that the effects on translation were underestimated and the effects on abundance were overestimated because a large percentage of targets mRNAs were translationally repressed, and stored without a poly(A) tail. To test this possibility, we measured the differences in total RNA levels irrespective of poly(A) tail for each gene between miR-124–transfected and mock-transfected cells. We found that the differences in RNA abundance between miR-124–transfected and mock-transfected cells as measured with unamplified total RNA were similar to those measured for amplified poly(A)-selected mRNA for miR-124 targets (Pearson *r* = 0.82, slope of least-squares regression fit in linear space = 0.82) (Figure S4). These data suggest that the apparent decrease in abundance of miR-124 target mRNAs results primarily from degradation rather than deadenylation alone.

### miR-124 Affects Both the Ribosome Occupancy and Ribosome Density of Hundreds of Targets

Many steps in protein synthesis have been proposed to be regulated by miRNAs. The proposed mechanisms include: (i) blocking initiation, e.g., by preventing eiF4F binding to mRNA caps or joining of the 40S and 60S ribosomal subunits; (ii) promoting poly(A) tail deadenylation, which can slow initiation by preventing interactions between the poly(A) tail and 5′-cap, and by increasing the rate of mRNA decay, which reduces the fraction of the mRNA's lifespan spent in the translated pool; (iii) promoting premature ribosome release during elongation; (iv) slowing translation elongation; (v) promoting cotranslational proteolysis; and (vi) concerted slowing of initiation and elongation [7],[10],[30],[34]–[37],[45],[51],[52],[54]–[62]. The first four proposed mechanisms make specific predictions about the effects of miRNAs on the ribosome occupancy and ribosome density of targets. Proposed mechanisms (i), (ii), and (iii) predict that both occupancy and density will decrease; mechanism (iv) predicts that ribosome density will increase as a function of the extent to which elongation is slowed. In contrast, proposed mechanism (v) does not predict that ribosome occupancy or ribosome density will change, and the effects on ribosome occupancy and ribosome density in mechanism (vi) depend on the relative effects of the miRNA on the two steps.

**Figure 5:**
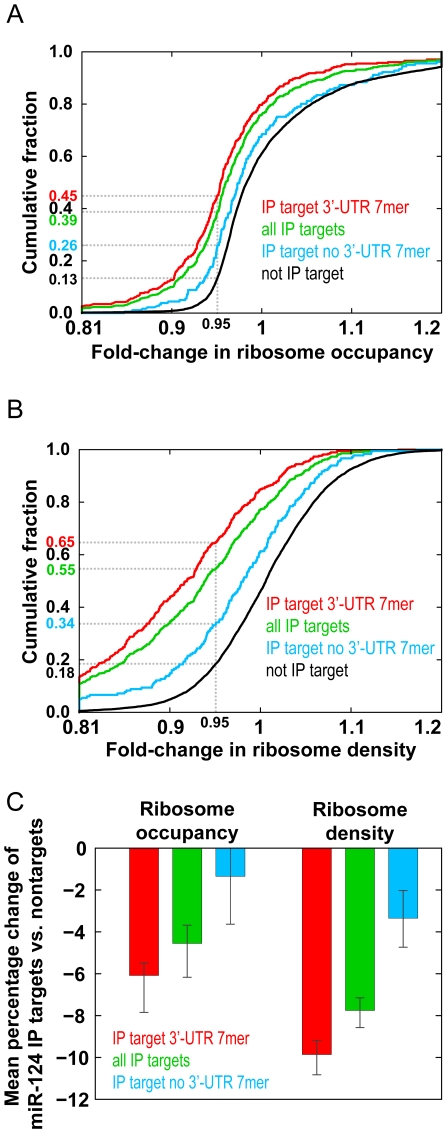
miR-124 Ago IP targets decrease in ribosome occupancy and ribosome density due to the presence of miR-124. (A) Cumulative distribution of the change in ribosome occupancy following transfection with miR-124, compared to mock. This analysis compares miR-124 Ago IP targets (1% local FDR) (green), IP targets with at least one 3′-UTR 7 mer seed match (red), IP targets that lacked a 3′-UTR 7mer seed match (blue), and nontargets (black). Changes in ribosome occupancy of miR-124 Ago IP targets were greater than those for nontargets (*p*<10^−31^). (B) Cumulative distribution of the change in ribosome density following transfection with miR-124 compared to mock. This analysis compares miR-124 Ago IP targets (1% local FDR) (green), IP targets with at least one 3′-UTR 7 mer seed match (red), IP targets that lacked a 3′-UTR 7 mer seed match (blue), and nontargets (black). Changes in ribosome density of miR-124 Ago IP targets were greater than those for nontargets (*p*<10^−51^). (C) Bar plot of the average change in ribosome occupancy (left) and ribosome density (right) following transfection with miR-124 of all miR-124 Ago IP targets (green), IP targets with at least one 3′-UTR 7mer seed match (red), IP targets that lacked a 3′-UTR 7mer seed match (blue). The average change in ribosome occupancy and ribosome density of targets was calculated by subtracting the average change of nontargets for the ribosome occupancy and ribosome density measurements following transfection with miR-124. The error bars represent 95% confidence intervals in the mean difference estimated by bootstrap analysis.

We tested these predictions by comparing ribosome occupancy and density profiles of mRNAs from miR-124 and from mock-transfected cells. We found that miR-124 Ago IP targets were much more likely than nontarget mRNAs to exhibit both reduced ribosome occupancy (Figure 5A) (*p*<10^−31^, one-sided Kolmogorov-Smirnov test) and reduced ribosome density (Figure 5B) (*p*<10^−51^, one-sided Kolmogorov-Smirnov test) following miR-124 transfection. Thirty-nine percent of miR-124 Ago IP targets decreased at least 5% in ribosome occupancy, compared to 13% of nontargets; 55% of miR-124 Ago IP targets decreased at least 5% in ribosome density, compared to 18% of nontargets. On average, the ribosome occupancy of miR-124 Ago IP targets decreased by 4%, and their ribosome density decreased by 8% (Figure 5C, green bars). We hypothesized that mRNAs with fewer associated ribosomes might exhibit larger changes in ribosome occupancy as a result of the increased likelihood of losing all ribosomes. In support of this hypothesis, on average, all ten miR-124 target mRNAs with ribosome occupancy changes greater than 20% had significantly shorter coding sequences and fewer bound ribosomes than mRNAs that changed less than 20% (*p* = 0.0003, one-sided Mann-Whitney test) (Figure S5A).

The effects on ribosome occupancy and ribosome density were significantly larger for miR-124 Ago IP targets that contain at least one 3′-UTR 7mer seed match (45% and 65% decreased at least 5% in ribosome occupancy and ribosome density, respectively), compared to miR-124 Ago IP targets that lack a 3′-UTR 7mer seed match (26% and 34% decreased at least 5% in ribosome occupancy and ribosome density, respectively), providing direct evidence for the general importance of 3′-UTR seed matches for miRNA-mediated translational repression of endogenous mRNAs [16],[17].

The observed effects on ribosome occupancy and density could, in principle, be the result of multiple independent regulatory mechanisms. For instance, the decrease in ribosome occupancy and density could be a result of mechanisms (i), (ii), and (iii). If however, the effects on ribosome occupancy and ribosome density were due to the same regulatory mechanism, we would expect a large overlap between mRNAs that show appreciable decreases in ribosome occupancy and ribosome density in the presence of miR-124. Indeed, 77% of miR-124 IP targets that decreased at least 5% in ribosome occupancy also decreased at least 5% in ribosome density (30% of all miR-124 IP targets decreased at least 5% in both ribosome occupancy and ribosome density compared to 2% of nontargets), which is significantly more than expected by chance (*p*<10^−18^, hypergeometric distribution). There was also a modest, but highly significant, correlation between changes in ribosome occupancy and ribosome density of miR-124 Ago IP targets (Spearman *r* = 0.45, *p*<10^−25^) (Figure S6A), although many mRNAs appeared to differentially change in either ribosome occupancy or ribosome density (some miR-124 mRNA targets even appeared to increase appreciably in ribosome occupancy; Figure S6 and Text S3). These results are consistent with the effects on ribosome occupancy and ribosome density arising from the same regulatory mechanism.

If miR-124 induced ribosome drop-off (mechanism (iii)) stochastically along the coding sequence, the change in ribosome density would be exponentially related to the length of the coding sequence. To test this, we plotted the change in ribosome density as a function of mRNA length for miR-124 IP targets and found that although they are correlated (Spearman *r* = 0.30), it is highly unlikely there is a first-order exponential relationship between the change in density and the length of the mRNA's coding sequence (*p*<10^−211^, F-test with the null hypothesis that the observed change in density fits the predicted change in density from an exponential least-squares fit) (Figure S5B). Thus, if ribosome drop-off is the predominant mode of miR-124 regulation, it occurs preferentially near the translation start site.

The observation that many miR-124 targets decreased in both ribosome occupancy and ribosome density after transfection with miR-124 is consistent with regulation of translation initiation (mechanisms (i) or (ii)) or ribosome drop-off preferentially near the translation start site (mechanism (iii)) by miR-124 and suggests that slowed elongation (model (iv)) is not the predominant mode of regulation of translation by miR-124 under these conditions. Without measurements of the actual effects on protein synthesis, these results, however, do not rule out the possibility that miR-124 also induces cotranslational proteolysis (v) or coordinately represses translation initiation and translation elongation (vi), resulting in modest decreases in ribosome occupancy and ribosome density, but large effects on protein synthesis.

### The Effects of miR-124 Transfection on Protein Products of miR-124 Targets

To analyze the overall effect of the observed decreases in mRNA abundance and translation on protein production, we calculated the estimated change in protein synthesis as:
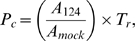
(2)where the estimated change in protein synthesis (*Pc*) can then be derived by multiplying the change in mRNA abundance by the estimated change in translation rate (*Tr*). The change in relative mRNA abundance is calculated by dividing relative mRNA abundance values from miR-124 transfection experiments by values from the mock condition 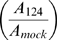. Although the overall effect on predicted protein production was on average quite modest (∼2-fold decrease compared to nontargets), for a small fraction of miR-124 targets, the predicted changes in protein production were fairly large; 45 of the 560 identified miR-124 targets were predicted to have a decrease of at least 4-fold in protein production 12 h after miR-124 transfection. A disproportionate fraction of the most significantly affected mRNAs encoded proteins associated with membrane compartments (28, *p* = 0.001), including endoplasmic reticulum (seven) and plasma membrane (nine); these mRNAs are likely to be translated on the rough endoplasmic reticulum. A similar observation was reported with different miRNAs in a recent study [17]. These results suggest that mRNAs that are translated on the rough endoplasmic reticulum might be particularly susceptible to miRNA-mediated regulation, possibly while stalled prior to engagement with the endoplasmic reticulum [87].

**Figure 6:**
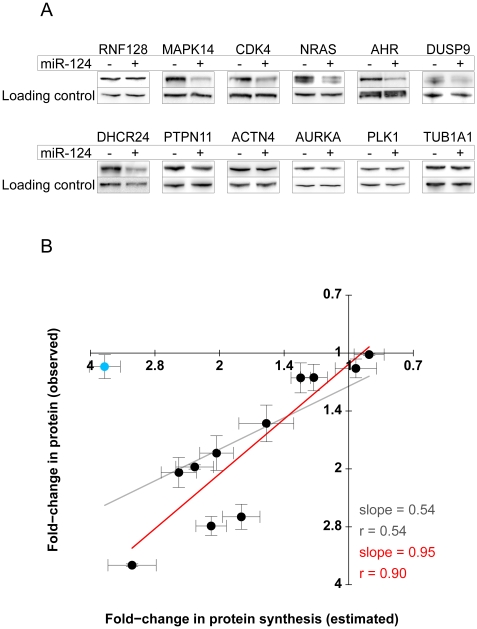
The effect of miR-124 transfection on protein production of miR-124 targets. (A) Western blots of 12 proteins encoded by mRNAs highly enriched in miR-124 Ago IPs from mock-transfected cells (−) and miR-124 transfected cells (+). The bottom bands are loading controls. The proteins are arranged according to decreasing estimated fold-change in protein synthesis from left to right. (B) Scatterplot between estimated changes in protein synthesis (*x*-axis) and observed changes in protein levels (*y*-axis) from Western blots. The gray line is a least-squares linear regression fit of all 12 proteins, and the red line is a least-squares fit of 11 proteins, excluding RNF128 (upper left protein, shown in blue).

To test whether our estimated changes in protein synthesis predict actual changes in protein abundance, we measured changes in protein abundance of a diverse set of proteins encoded by mRNAs that are highly enriched in miR-124 Ago IPs by Western blot analysis based on the availability of reliable antibodies. We chose 14 proteins encoded by mRNAs that are highly enriched in miR-124 Ago IPs, with predicted decreases in protein synthesis ranging from no change to 3-fold (Table S1). We collected cell lysates 60 h (four to five cell divisions) after miR-124 or mock-transfection to reduce the likelihood of underestimating the change in protein synthesis for long-lived proteins. Twelve of the 14 antibodies detected bands at the predicted molecular weight (Figure 6A). We observed a significant correlation between the estimated changes in protein synthesis (Figure 6B, *x*-axis) and the measured changes in protein levels (Figure 6B, *y*-axis) in response to miR-124 transfection (Spearman *r* = 0.54, *p* = 0.07, slope of least-squares regression fit = 0.54, grey line in Figure 6B), with one exception. Only RNF128, with a predicted 3.7-fold reduction in protein synthesis, drastically disagreed with our measured decrease of 1.2-fold reduction. It is possible that the discordance in RNF128 protein levels is due to posttranslational autoregulation, which is common among ring finger proteins [88]–[90]. After excluding RNF128 from analyses, there is a strong concordance between the two measurements (Spearman *r* = 0.90, *p* = 0.0001, slope = 0.95; red line, Figure 6B) for the remaining 11 proteins. The high correlation and the fact that the slope of the best-fit line excluding RNF128 is close to one, suggests that miR-124–induced changes in transcript abundance and translation rate can almost completely account for the changes in abundance of the targeted proteins. Thus, cotranslation proteolysis (proposal (v)) and coordinate repression of initiation and elongation (proposal (vi)) are unlikely to play more than a minor role in miR-124 regulation under these conditions.

### Concordant Changes in Abundance and Translation of mRNAs Targeted by miR-124 Suggests That These Two Regulatory Outcomes Are Functionally Linked

Multiple distinct miRNA regulatory pathways have been proposed, such that translational repression and mRNA degradation can be regulated independently, and these two regulatory consequences are differentially affected by specific features of miRNA–mRNA interaction [34],[37],[67],[71],[91]. The relative magnitude of effects on translation and decay of targeted mRNAs might be influenced by the sequence context of the miRNA–mRNA interaction and the particular suite of RNA-binding proteins associated with the mRNA [38],[43],[65],[92]. If the balance between effects on translation and effects on decay were influenced in a gene-specific way by features of the mRNA, we would expect that some targets of miR-124 would have relatively large changes in translation with little change in mRNA abundance or vice versa. If, however, miRNA–mRNA interactions act through a single dominant regulatory pathway that affects both translation and decay, we would expect a strong correlation between the changes in abundance and translation of mRNA targets of miR-124.

**Figure 7:**
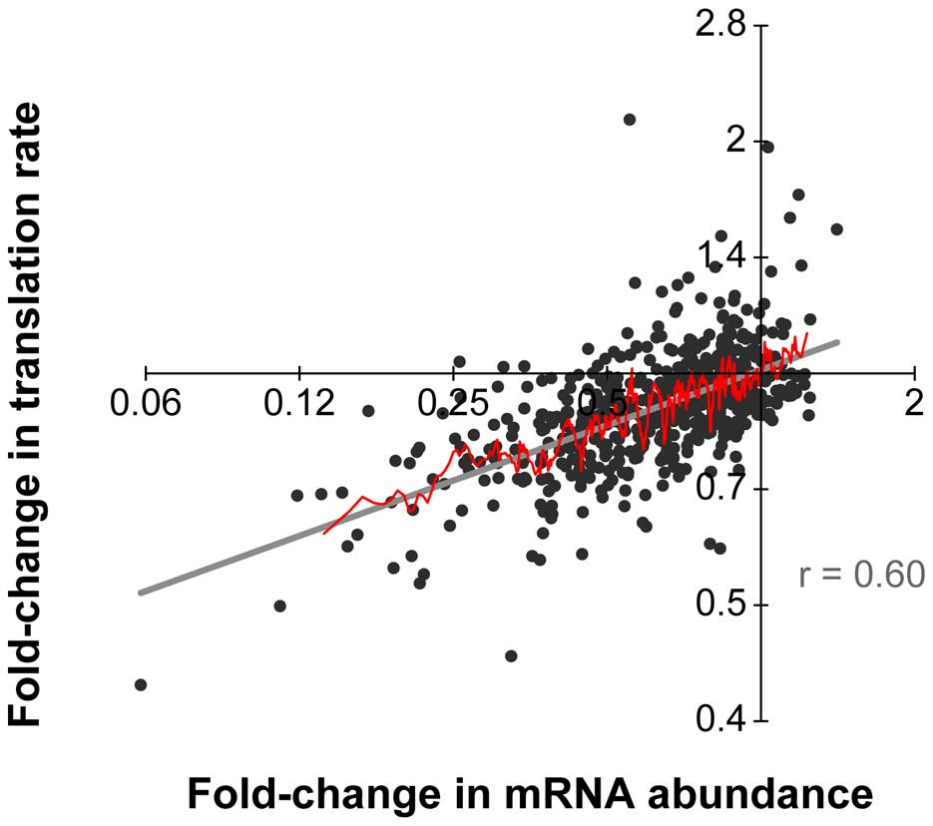
Concordant changes in mRNA abundance and translation of miR-124 Ago IP targets. Scatterplot between changes in mRNA abundance (*x*-axis) and the estimated translation rate (*y*-axis) for miR-124 Ago IP targets following transfection with miR-124 compared to mock. The gray line is a least-squares linear regression fit of the data, and the red line is a moving average plot (window of 10). The slope of the least-squares fit of the data is 0.24 (in linear space, 0.36), and the Pearson correlation is 0.60.

We compared the changes in mRNA abundance (Figure 7, *x*-axis) to apparent changes in translation rate (Figure 7, *y*-axis) for miR-124 Ago IP targets following miR-124 transfection. There was strong correlation between these two regulatory effects (Pearson *r* = 0.60, see Text S4 and Figure S7 for estimates of significance of the correlation), and we found no subpopulation of mRNAs whose translation was appreciably diminished without corresponding changes in mRNA abundance, and few mRNAs whose abundance changed significantly without a corresponding change in translation. To test whether the apparent correlation might be driven solely by mRNAs with the largest measured changes in abundance and translation, we calculated the average changes in mRNA abundance and translation in moving windows of ten mRNAs ranked by their change in mRNA abundance. As shown in Figure 7 (red curve), we found a persistent, nearly monotonic, relationship between changes in mRNA abundance and translation that closely matches the least-squares fit of all the data (Pearson *r* = 0.91). We obtained similar results when we analyzed miR-124 Ago IP targets with 7mer 3′-UTR seed matches and those that lacked a 7mer 3′-UTR seed match (Figure S8), although the correlation was stronger for targets with 7mer 3′-UTR seed matches (*r* = 0.60 versus 0.42).

The correlation between changes in mRNA abundance and estimated translation rate, and the absence of a subgroup of mRNAs regulated at the translational level without corresponding effects on abundance, is consistent with a model in which these two regulatory programs are functionally linked. Although there was a measurable decrease in mRNA abundance for almost all miR-124 targets that significantly decreased in translation, only about half of the targets that decreased in mRNA abundance registered a measurable reduction in translation. It is possible that some mRNA targets are degraded without any appreciable effect on translation (e.g., the mRNAs are degraded while still associated with ribosomes) or that translation of these mRNAs is indirectly stimulated in response to miR-124, resulting in no apparent effect on translation at the time we performed translation assays. Alternatively, as the changes in translation tended to be smaller than the changes in mRNA abundance, we may have been unable to accurately measure the small effects on translation of many targets.

### Changes in Abundance and Translation of miR-124 Ago IP Targets with Seed Matches in 3′-UTRs, Coding Sequences, and 5′-UTRs

Although most functional microRNA seed matches are located in 3′-UTRs as judged by mRNA expression data, phylogenetic conservation analysis, Ago IPs, and reporter studies, some sites in coding sequences and 5′-UTRs can also confer regulation by miRNAs [4],[16],[17],[59],[70],[71],[78],[79],[93]–[95]. The 560 high-confidence miR-124 Ago IP targets for which we obtained high-quality measurements in expression and translation analyses were strongly enriched for mRNAs that contained miR-124 seed matches in 3′-UTRs and coding sequences (Figure 1B), but they were also significantly, albeit weakly, enriched, for seed matches in 5′-UTRs (16, *p* = 0.009).

We compared the effectiveness of 7mer seed matches in the 3′-UTR, coding sequence, and 5′-UTR, and 6mer seed matches in the 3′-UTR in effecting changes in mRNA abundance and estimated translation rate. We found that both the abundance and translation rate of IP targets, regardless of the location of seed matches, decreased relative to nontarget mRNAs in miR-124 transfected cells compared to mock-transfected cells (Figure S9). The estimated effects on protein production were greatest for mRNAs with 7mer seed matches in the 3′-UTR, consistent with previous studies reporting that 3′-UTR seed matches confer the highest degree of regulation [11],[70],[71],[93]. Changes in mRNA abundance were significantly greater than changes in translation for miR-124 Ago IP targets with 3′-UTR and coding sequence seed matches (Figure S9). IP targets that did not contain any 6mer seed matches were also significantly more likely to decrease in mRNA abundance than nontargets (Figure S9), which suggests that many of these mRNAs are specifically recruited to Agos by miR-124 and regulated by miR-124, even though they do not appear to have canonical recognition elements.

### Efficiency of Recruitment to Argonautes by miR-124 Seed Matches Correlates with Effects on Both mRNA Abundance and Translation

The extent to which each of the thousands of genes expressed in a given mammalian cell is regulated by the suite of (often hundreds of) miRNAs expressed in that cell is not known. We reasoned that our Ago immunopurification strategy, by quantitatively measuring association of mRNAs with microRNA effector complexes, could serve as a direct readout of the potency of the regulatory effects of miRNAs on each mRNA. We compared the change in Ago IP enrichment following transfection with miR-124 to the estimated changes in protein production (Equation 2) for mRNAs with seed matches to miR-124 in their 3′-UTR or coding sequence (Figure S10). For mRNAs with 7mer or 8mer seed matches to miR-124 in their 3′-UTR, there was a strong negative correlation between the magnitude of their enrichment by the Ago IP and the estimated changes in production of the protein they encode (3′-UTR 7mer: Pearson *r* = −0.72, *p*<10^−192^; 3′-UTR 8mer: *r* = −0.72, *p*<10^−26^) (Figure S10A). For mRNAs with 7mer or 8mer seed matches to miR-124 in their coding sequences, but no 7mer seed matches in their 3′-UTRs, there was also a significant, albeit weaker, correlation (coding sequence 7mer: *r* = −0.39, *p*<10^−33^; coding sequence 8mer: *r* = −0.38, *p*<10^−4^) (Figure S10B). There was also a weak, but still significant, correlation between IP enrichment and the estimated change in protein production for mRNAs that lacked 7mer seed matches in their 3′-UTR or coding sequence or that lacked even 6mer seed matches in their 3′-UTR or coding sequence, respectively (3′-UTR 6mer: *r* = −0.40, *p*<10^−75^; no 3′-UTR or CDS 6mer: *r* = −0.23, *p*<10^−24^) (Figure S10C). Most of the mRNAs with 7mer or 8mer seed matches in their 3′-UTR or coding sequence that decreased significantly in protein production were enriched in the Ago IPs. Thus, Ago IP enrichment following transfection with a specific miRNA appears to be a good predictor of the corresponding effects on protein production. Because changes in mRNA abundance and translation following transfection of a specific miRNA are quantitatively smaller and less specific than their change in association with Agos, the IP method appears to be a more sensitive assay to identify the direct regulatory targets of specific miRNAs.

## Discussion

miRNAs regulate the posttranscriptional fates of most mammalian mRNAs, yet for endogenous mRNAs, the effects of miRNAs on translation, the steps in translation that are regulated by miRNAs, and the relationship between regulation of translation and mRNA decay by miRNAs have not been systematically explored. To address these effects and relationships, we determined the effect of a human miRNA, miR-124, on translation and abundance of hundreds of endogenous mRNAs that were recruited to Argonaute proteins in response to ectopic expression of miR-124 in HEK293T cells.

We developed a simple and economical method to quantitatively measure two key parameters of translation, ribosome occupancy and average ribosome density, on a genome-wide scale with single DNA microarray hybridizations for each (Figure 2). This method allowed us to address the effects of miR-124 on translation of endogenous mRNAs; it is also more broadly applicable to the study of translational regulation. In this initial application, we found many parallels between the translation programs in proliferating human embryonic kidney cells and *S. cerevisiae* (Figure 3), suggesting common features of translational programs in eukaryotes [80].

Direct identification of the mRNAs specifically recruited by miR-124 to Ago proteins, core components of miRNA-effector complexes, defined functional targets of this miRNA in this model system, providing a starting point for dissecting miRNA regulation [70]–[72],[96],[97]. mRNA expression profiling then allowed us to recognize the specific effects of miR-124 on the abundance of these targets.

Three major conclusions emerged from our studies: (i) miR-124 reduces translation and abundance of its mRNA targets over a broad range; changes in mRNA abundance accounted for ∼75% of the estimated effect on protein production; (ii) miR-124 predominantly targets translation at the initiation stage or stimulates ribosome drop-off preferentially near the translation start site; and (iii) miR-124–mediated regulation of translation and mRNA decay are correlated, indicating that most mRNAs are not differentially targeted for translational repression versus mRNA decay.

Transfection of miR-124 consistently reduced the translation and abundance of most of its several hundred high-confidence targets; the resulting decrease in translation averaged 12% and the decrease in target mRNA abundance averaged 35% (Figure 4). The observation that there were several mRNAs (CD164, VAMP3, and DNAJC1) that had about 10-fold reductions in mRNA levels (Figure S7), and the fact that 90% of control-transfected cells expressed the transfected GFP marker, suggests that more than 90% cells were transfected with functionally significant quantities of miR-124; thus the small magnitude of the effects on translation and abundance of most of the mRNA targets of miR-124 identified by Ago IP was not likely a result of poor transfection efficiency. The correlation between predicted changes in protein synthesis and observed changes in protein levels for 11 of 12 proteins following miR-124 transfection (Figure 6), suggests that our assays capture most (or all) of the effects of miR-124 on protein synthesis.

Although we need to be cautious in generalizing from these model systems, in these cells under the condition examined, miRNAs appears to modulate production for hundreds of proteins through joint regulation of target mRNA translation and stability over a strikingly large dynamic range. While the repressive effects on most targets were modest (1–3-fold), there were eight targets (DNAJC1, VAMP3, CD164, SYPL1, MAGT1, HADHB, ATP6V0E1, and SGMS2) that were substantially down-regulated with decreases in protein synthesis of 10-fold or greater. In addition, 45 targets were estimated to have greater than 4-fold changes in protein synthesis. Regardless of the magnitude of regulation, mRNA destabilization accounted for ∼75% of the change in estimated protein synthesis. This range of regulation is in good accord with previous studies with genetically characterized endogenous miRNAs as well as with studies introducing exogenous miRNAs introduced into human tissue culture [7],[9],[16],[17],[33]. However, our observation that miR-124 had only modest effects on the translation of hundreds of targets contrasts dramatically with several previous studies in which miRNAs reduced protein expression by 5–25-fold while only modestly decreasing mRNA levels (1.1–2-fold), suggesting substantial inhibitory effects on translation [37],[44],[61],[69],[91]. The previous studies, however, measured the effect of a specific miRNA on reporter constructs in which the 3′-UTRs of the encoded mRNAs were not derived from mammalian mRNAs, but were either short (∼250 nts) modified viral sequences or artificial. In contrast, mammalian mRNA 3′-UTRs tend to be much longer (on average ∼1,000 nts) and include regulatory sites for RNA-binding proteins and regulatory RNAs that influence mRNA localization, translation, and decay. The basis for the discrepancy in the results from these two experimental designs remains to be determined, and the answer is likely to provide useful mechanistic insights. One possibility is that mRNAs containing exogenous 3′-UTRs might have anomalously long mRNA half-lives that obscure the normal contribution of mRNA degradation to the miRNA-directed inhibition of protein expression. The large magnitude of effects observed in reporter-based assays, compared to what we and others have observed with endogenous mRNAs, is likely to be partially due to the multiple (four to eight) engineered miRNA binding sites in the reporter constructs used in those studies [37],[44],[61],[69],[91]. Further, these sites were in close proximity, and adjacent miRNA binding sites have been reported to act cooperatively [36],[93],[98]. Indeed, two studies that measured the effects of specific miRNAs on protein and mRNA levels of reporters with endogenous mammalian 3′-UTRs found more modest effects on translation, less than 2-fold on average [11],[72]. Moreover, the magnitude of the effects we observed on translation of the mRNAs targeted by miR-124 were in agreement with two recent studies that inferred the repressive effect of miRNAs on translation by measuring miRNA-mediated effects on mRNA and protein abundance [16],[17]. Those reports, based on directly measured changes in protein levels by quantitative mass spectrometry, concluded that the effects of miRNAs on translation were small (less than 2-fold for hundreds of target mRNAs).

Although we believe that our experimental design provided a good model of miRNA regulation as it normally operates in vivo, our results do represent the full range of possible regulatory consequences of miRNA–mRNA interactions. Our results suggest that miRNAs have a large dynamic range of effects on endogenous protein expression, achieved via regulation of both translation and mRNA abundance; this pattern is generally quite consistent with previous results from cells grown in culture and limited in vivo observations. However, in specific developmental or physiological programs, or for specific mRNAs, the effects on abundance and translation, as well as the apparent mode of translation regulation may differ from what we observed in this study [7],[33],[60],[62],[99]. Thus, the effects we observed for miR-124 targets after ectopically expressing the microRNA in Hek293T cells may not capture the full scope of regulation by miRNAs in their endogenous context; miR-124 is endogenously expressed in neuronal cells, and the regulatory effects of miR-124 interactions may be modulated by the physiological demands of the cell and the specific suite of specific RNA-binding proteins and regulatory RNAs that also associate with miR-124 target mRNAs.

miR-124 negatively affected both the ribosome occupancy and ribosome density of hundreds of its targets (Figure 5). These parallel effects, combined with the close match between changes in protein synthesis predicted from miRNA-induced effects on mRNA abundance and translation and changes in protein levels for 11 of 12 proteins, suggest that the step in translation principally targeted by miR-124 and presumably other miRNAs is initiation or elongation processivity near the translation start site. We favor the initiation model because it is in accord with several previous studies that focused on one or a few mRNAs [10],[44],[52],[54]–[58], and there is a paucity of empirical evidence supporting ribosome drop-off, which predicts that ribosome density of miRNA-regulated mRNAs declines between the 5′- and 3′-ends of the coding sequence and that there should be an overrepresentation of incompletely synthesized N-terminal nascent polypeptides [61].

The small apparent magnitude of the effects on translation initiation, combined with the strong correlation between changes in translation and mRNA abundance, can be explained by a model in which repression of translational by miR-124 rapidly leads to mRNA decay. Such a model would explain why observable effects on translation appear to be smaller than the changes in mRNA abundance: if mRNAs whose translation is inhibited are quickly destroyed, their diminished translation would not be detected in our translation assay. There is already compelling evidence that translational repression and mRNA decay are linked [39],[100]–[112]. Our observation that an overwhelming majority of polyadenylated mRNAs are associated with ribosomes in HEK293T cells may be a manifestation of this relationship (Figure 3A). Thus, miRNA-mediated inhibition of translation may be linked to a general system for removal of the mRNA from the translational pool, involving recruitment to P-bodies and subsequent destruction [39]–[44]. Regulated decoupling of miRNA-mediated translation repression and mRNA decay would then allow organisms to tilt the balance of effects in favor of translational repression during physiological and developmental conditions where mRNA destruction is a disadvantage [99]. Our results are also consistent with a model in which miRNA-mediated regulation of translation and mRNA decay are functionally independent, but are similarly controlled by the same *cis*-elements. Determining whether the concordant regulation of translation and mRNA abundance represents a mechanistic coupling of miRNA-mediated regulation of translation and mRNA decay, and understanding the molecular links between these two regulatory consequences of miRNA–mRNA interactions are important goals for future investigation.

## Materials and Methods

### Plasmids and Oligonucleotides

miR-124 siRNA:

sense: 5′-UAA GGC ACG CGG UGA AUG CCA-3′

antisense: 5′-GCA UUC ACC GCG UGC CUU AAU-3′

### Cell Culture and Transfection

HEK293T cells were obtained from ATCC (Cat# CRL-11268) and grown in Dulbecco's modified Eagle's medium (DMEM) (Invitrogen) with 10% fetal bovine serum (Invitrogen) and supplemented with 100 U/ml penicillin, 100 mg/ml streptomycin, and 4 mM glutamine at 37°C and 5% CO_2_. Transfections of HEK293T cells were carried out with calcium phosphate. Cells were plated in 15-cm dishes 12 h prior to transfection at 2×10^5^ cells per ml (25 ml total). We made mock-transfection mixture (1/10 volume of growth medium) by diluting 152 µl of 2 M CaCl_2_ into 1.25 ml of nuclease-free H_2_O and then slowly adding this solution to 1.25 ml of 2× HBS (50 mM Hepes [pH 7.1], 280 mM NaCl, 1.5 mM Na_2_HPO_4_). After 1 min, the mixture was added to a 15-cm plate at a medium pace. Transfections with miR-124 oligonucleotides were performed analogously with 30 nM of oligonucleotides in 2.5 ml of transfection mixture.

### Preparation of Beads for Immunopurifications

Ago-specific 4f9 hybridoma was grown in suspension and adapted to 10% FBS-enriched DMEM [73]. We purified the antibody by passing supernatant from 1 l of culture over a 5-ml protein L-agarose column (Pierce Cat# 89929) as per the vendor's instructions. Eluent fractions were pooled and dialyzed into PBS with Slide-A-Lyzer Dialysis Cassettes (Pierce Cat# 66382). We then biotinylated the purified 4f9 antibody with No-Weigh NHS-PEG_4_-Biotin Microtubes (Pierce Cat# 21329). We quantified biotinylation with EZ Biotin Quantitation Kit (Pierce Cat# 28005). Biotinylated 4f9 antibody was aliquoted and stored at −80°C until use. For Ago immunopurifications, we coupled biotinylated 4f9 antibody to DYNAL Dynabeads M-280 Streptavidin magnetic beads (Invitrogen Cat# 112-06D) (50 µg of antibody per ml of beads) as per vendor's instructions and stored the coupled beads at 4°C for up to 1 wk before use.

### Immunoaffinity Purifications

Twelve hours after transfection, we washed each 15-cm plate once with phosphate-buffered saline (usually two plates were used per IP), then added 1 ml of 4°C lysis buffer (150 mM KCl, 25 mM Tris-HCl [pH 7.4], 5 mM Na-EDTA [pH 8.0], 0.5% Nonidet P-40, 0.5 mM DTT, 10 µl protease inhibitor cocktail [Pierce Cat# 78437], 100 U/ml SUPERase•In [Ambion Cat# AM2694]). Following a 30-min incubation at 4°C, we scraped the plates, combined the lysates, and then spun them at 4°C for 30 min at 14,000 RPM in a microcentrifuge. We collected the supernatant and filtered it through a 0.45-µm syringe filter. We froze an aliquot of lysate in liquid nitrogen for reference RNA isolation. We then added 0.22 mg/ml heparin to the lysate. We mixed the lysate with 2.5 mg of Dynal M-280 Streptavidin beads (250 µl from original storage solution) coupled to biotinylated 4F9 ago antibody (∼12.5 µg), which we equilibrated immediately prior to use by washing twice with 1 ml of lysis buffer. We incubated the beads with the lysate for 2 h at 4°C and then washed the beads twice with 1.25 ml of ice-cold lysis buffer for 5 min each. Five percent of the beads were frozen for SDS PAGE analysis after the second wash. RNA was extracted directly from the remaining beads using lysis buffer from Invitrogen's Micro-to-Midi kit (Invitrogen Cat# 12183-018). We purified RNA from the lysate and RNA extracted from the beads with the Micro-to-Midi kit as per vender's instructions, except that the percentage isopropanol used for binding to the column was 70%, instead of 33%, to promote the binding of small RNAs.

### Western Blots

Sixty hours after transfection, HEK293T cell lysate was prepared using the same protocol for immunoaffinity purifications. The concentration of protein in each sample was quantified using the BCA assay (Pierce Cat#23255). For SDS-PAGE separation, 25 µg of protein from each sample was used. Protein was then transferred on to a polyvinylidene fluoride (PVDF) membrane for detection with the following specific antibodies: DUSP9 (Abcam Cat# ab54941-100); PTPN11 (Bethyl Laboraties Cat# a301–544a); ITGB1 (BD Transduction Laboratories Cat# 610467); AURKA, DHCR24, MAPK14, and PLK1 (Cell Signaling Cat# 4718, 2033s, 9212, and 4513, respectively); AHR, ACTN4, CDK4, RNF128, NRAS, and PTBP2 (Santa Cruz Biotechnology Cat# sc-5579, sc-17829, sc-260, sc-101967, sc-519, and sc-101183, respectively); and TUBA1A (Sigma Cat# 096K4777). GAPDH and TUBB1 (Abcam Cat# ab9484, ab6046) were used as loading controls to check for lane-specific differences from loading, transfer, and detection errors. Protein bands were quantified using the BioRad Quantity One software package.

### Preparation of Cell Extracts for Translation Profiling

For translation experiments, two 15-cm dishes of cells (per condition) were seeded, grown, and transfected as described above. Twelve hours after transfection, high-purity cyclohexamide (Calbiochem Cat# 239764) was added at a final concentration of 0.1 mg/ml directly into growth media, and the plate was agitated for 1 min at room temperature. Plates were then placed on ice and washed twice with 10 ml of ice-cold buffer A (20 mM Tris [pH 8.0], 140 mM KCl, 5 mM MgCl_2_, 0.1 mg/ml cycloheximide). After the second wash was aspirated, the plates were tilted and left for 1 min on ice to facilitate removal of excess liquid. Each plate was then washed 1× with 2 ml of ice-cold buffer A that contained 0.22 mg/ml of heparin. After removal of excess liquid, cells were scraped from each dish and collected in a 1.5-ml microcentrifuge tube on ice. Each plate typically yielded about 300 µl (for 600 µl total) of cells and residual buffer. This mixture was then brought to 1× protease inhibitor cocktail (Pierce Cat# 78437), 100 U/ml SUPERASin, and 0.5 mM DTT. To lyse the cells, the cell-buffer mixture was brought to 0.1% Brij 58 (Sigma Aldrich Cat# P5884-100G) and 0.1% sodium deoxycholate (Sigma Aldrich Cat# D6750-100G) and vortexed for 1 min. The lysate was subsequently spun at 3,500 rpm in a microcentrifuge for 5 min at 4°C. Supernatant was collected in a fresh tube and spun at 9,500 rpm in a microcentrifuge for 5 min at 4°C. Supernatant was collected, flash frozen in liquid nitrogen, and then stored at −80°C until use.

### Sucrose Gradient Preparation

Sucrose gradients were prepared using the Gradient Master (Biocomp) according to the manufacturer's suggestions. Five percent and 60% (w/v) sucrose solutions were prepared by dissolving sucrose in Gradient Buffer (20 mM Tris-HCl [pH 8.0], 140 mM KCl, 5 mM MgCl_2_, 0.5 mM DTT, 0.1 mg/ml cycloheximide) at room temperature. The 60% solution was dispensed into an SW41 ultracentrifuge tube through a cannula underneath the 5% solution. Using an 11-step program (Biocomp, SW41 SHORT SUCR 5–50 11), the two solutions were mixed on the Gradient Master to form a linear gradient. After preparation, gradients were placed in chilled SW41 ultracentrifuge buckets and equilibrated for several hours at 4°C.

### Sucrose Gradient Velocity Sedimentation

Immediately before centrifugation, 300 µl of lysate (∼300 µg of total RNA) was transferred to the surface of the gradient. Gradients were centrifuged at 41,000 rpm (RCFave = 207,000) for 70 min at 4°C using a SW41 rotor and then stored at 4°C until fractionation. The Gradient Station (Biocomp) trumpet tip was pushed into the ultracentrifuge tube at a rate of 0.17 mm per second. Fractions (550 µl) were collected into a 96-well plate containing 600 µl of lysis solution (Invitrogen) using a fraction collector (Teledyne-Isco). The absorbance of the gradient at 260 nm was measured during fractionation using a UV6 system (Teledyne-Isco).

### Gradient Encoding

Immediately after fractionation, a unique set of four to five polyadenylate-tailed control RNAs, corresponding to *Methanococcus jannaschii* mRNAs that do not share significant identify to sequences in the human genome, were added at 100 pg each to fractions that contained the 80S ribosome and polysomes (Table S2). The solution was mixed well by inverting the plate several times, and liquid was collected in the well bottom by a brief centrifugation. A Precision XS liquid handler (BioTek Intruments) was used to transfer a defined volume of each of the fractions to one of four tubes (Fisher Cat# 14-959-11B) (Table S2); the solutions in each tube are referred to as pool “A,” “B,” “C,” and “D,” respectively. Upon completion of liquid handling, eight additional control RNAs (Ambion Cat# 1780) (Table S1) were added to each pool, and the pools were stored at −20°C.

Pools A–D were thawed at room temperature for 30 min. Two volumes of isopropanol was added to each pool, and the RNA in each pool was isolated from the mixture using the Micro-to-Midi RNA isolation kit (Invitrogen Cat# 12183-018).

### DNA Microarray Production and Prehybridization Processing

HEEBO oligonucleotide microarrays were printed on epoxysilane-coated glass (Schott Nexterion E) by the Stanford Functional Genomic Facility. The HEEBO microarrays contain ∼45,000 70-mer oligonucleotide probes, representing ∼30,000 unique genes. A detailed description of this probe set can be found at (http://microarray.org/sfgf/heebo.do) [113].

Prior to hybridization, slides were first incubated in a humidity chamber (Sigma Cat# H6644) containing 0.5× SSC (1× SSC = 150 mM NaCl, 15 mM sodium citrate [pH 7.0]) for 30 min at room temperature. Slides were snap-dried at 70–80°C on an inverted heat block. The free epoxysilane groups were blocked by incubation with 1M Tris-HCl (pH 9.0), 100 mM ethanolamine, and 0.1% SDS for 20 min at 50°C. Slides were washed twice for 1 min each with 400 ml of water, and then dried by centrifugation. Slides were used the same day.

### DNA Microarray Sample Preparation, Hybridization, and Washing

Amplified RNA was used for most DNA microarray experiments. Poly-adenylated RNAs were amplified in the presence of aminoallyl-UTP with Amino Allyl MessageAmp II aRNA kit (Ambion Cat# 1753). For mRNA expression experiments, universal reference RNA was used as an internal standard to enable reliable comparison of relative transcript levels in multiple samples (Stratagene Cat# 740000). Amplified RNA (3–10 µg) was fluorescently labeled with NHS-monoester Cy5 or Cy3 (GE HealthSciences Cat# RPN5661). Dye-labeled RNA was fragmented (Ambion Cat# 8740), then diluted into in a 50-µl solution containing 3× SSC, 25 mM Hepes-NaOH (pH 7.0), 20 µg of human Cot-1 DNA (Invitrogen Cat# 15279011), 20 µg of poly(A) RNA (Sigma Cat# P9403), 25 µg of yeast tRNA (Invitrogen Cat# 15401029), and 0.3% SDS. The sample was incubated at 70°C for 5 min, spun at 14,000 rpm for 10 min in a microcentrifuge, then hybridized at 65°C using the MAUI hybridization system (BioMicro) for 12–16 h. For translation experiments, amplified RNA from pools A and C was fluorescently labeled with NHS-monoester Cy5, and RNA from pools B and D was fluorescently labeled with NHS-monoester Cy3. Amplified RNA from pools A and B were comparatively hybridized to a DNA microarray to obtain the average ribosome density, and amplified RNA from pools C and D were comparatively hybridized to a DNA microarray to measure ribosome occupancy.

To compare total RNA levels in miR-124 and mock-transfected cells (Figure S3), 5–10 µg of total RNA from miR-124–transfected cells or mock-transfected cells or universal reference RNA (Stratagene Cat# 740000) was reverse transcribed with Superscript III (Invitrogen Cat# 18080085) in the presence of aminoallul-dUTP 5-(3-aminoallyl)-dUTP (Ambion Cat# AM8439) and natural dNTPs (GE Healthsciences Cat# US77212) with 10 µg of N9 primer (Invitrogen). Subsequently, amino-allyl–containing cDNAs from miR-124 and mock-transfected cells were covalently linked to Cy5 NHS-monoesters, and universal reference cDNA was covalently linked to Cy3 NHS-monoesters (GE HealthSciences Cat# RPN5661). Cy5- and Cy3-labeled cDNAs were mixed and diluted into 50 µl of solution containing 3× SSC, 25 mM Hepes-NaOH (pH 7.0), 20 µg of human Cot-1 DNA (Invitrogen Cat# 15279011), 20 µg of poly(A) RNA (Sigma Cat# P9403), 25 µg of yeast tRNA (Invitrogen Cat# 15401029), and 0.3% SDS. The sample was incubated at 95°C for 2 min, spun at 14,000 rpm for 10 min in a microcentrifuge, then hybridized at 65°C for 12–16 h.

Following hybridization, microarrays were washed in a series of four solutions containing 400 ml of 2× SSC with 0.05% SDS, 20058 SSC, 1× SSC, and 0.2× SSC, respectively. The first wash was performed for 5 min at 65°C. The subsequent washes were performed at room temperature for 2 min each. Following the last wash, the microarrays were dried by centrifugation in a low-ozone environment (<5 ppb) to prevent destruction of Cy dyes [114]. Once dry, the microarrays were kept in a low-ozone environment during storage and scanning (see http://cmgm.stanford.edu/pbrown/protocols/index.html).

### Scanning and Data Processing

Microarrays were scanned using AxonScanner 4000B (Molecular Devices). PMT levels were autoadjusted to achieve 0.1–0.25% pixel saturation. Each element was located and analyzed using SpotReader (Niles Scientific) and GenePix Pro 6.0 (Molecular Devices). For IP and mRNA expression experiments, the microarrays were submitted to the Stanford Microarray Database for further analysis [115]. Data were filtered to exclude elements that did not have one of the following: a regression correlation of ≥0.7 between Cy5 and Cy3 signal over the pixels compromising the array element, or an intensity/background ratio of ≥3 in at least one channel.

Ribosome density (pool A versus B) and ribosome occupancy (pool C versus D) measurements were normalized using exogenous doping control RNAs to correct for experimental variation between the two pools from RNA isolation, labeling efficiency, and scanning levels. In most cases, oligonucleotides that were designed to measure the exogenous doping control RNAs were represented multiple times on the DNA microarray (up to eight) and printed from different plates with different print tips. For ribosome occupancy experiments, the measured Cy5/Cy3 ratios of features on the microarray that correspond to the eight RNA controls added to pools C and D were fit to their expected Cy5/Cy3 ratios using least-squares linear regression in the statistical computing program R. The slope and *y*-intercept were used to rescale the measured Cy5 value of all other features on the DNA microarray. The ribosome occupancy for each RNA was then calculated as the corrected Cy5 intensity/(corrected Cy5 intensity + Cy3 intensity) (Figure S3C).

To calculate the average number of ribosomes bound to each mRNA, the measured Cy5/Cy3 ratios of features on the microarray that correspond to the 85 *M. jannaschii* doping control RNAs that were added to fractions that contained ribosomes pools was fit to their expected Cy5/Cy3 ratios using least-squares linear regression. The slope and *y*-intercept were used to rescale the measured Cy5 value of all other features on the DNA microarray (Figure S3B).

The average ribosome density was calculated by dividing the average ribosome number by coding sequence length and then multiplying the result by 100 to give density per 100 nts. The average ribosome number was calculated using two relationships. For each ribosome peak in the profile, the distance traveled from the start point was determined. In all gradients, we could clearly resolve peaks for up to seven bound ribosomes, and we used least-squares regression to relate the ribosome peaks 1–7 to their distance traveled in the gradient according to the following equation:
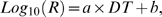
(3)where *R* represents the number of ribosomes bound, *DT* represents the distance traveled, and *a* and *b* are the slope and *y*-intercept, respectively. We then recorded the distance between the midpoint of each fraction to the start of the profile for each of the 15 ribosome-bound fractions and used the slope and *y*-intercept from Equation 3 to calculate the number of ribosomes at each fraction midpoint. The gradient encoding ratio at each fraction midpoint is the result of differential partitioning of each fraction in a predetermined manner into the heavy and light pools, and the ratio can be related to the ribosome number at each fraction midpoint using least-squares linear regression as described by Equation 4:
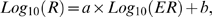
(4)where *R* represents the average ribosome number, and *ER* represents the encoding ratio. Finally, the average number of ribosomes bound for each gene's mRNAs was calculated using the slope and *y*-intercept from Equation 4.

Prior to normalization, spots with intensity/background of less than 1.5 for either Cy3 or Cy5 channel were filtered.

The microarray data are available from Gene Expression Omnibus (GEO) (http://www.ncbi.nlm.nih.gov/geo/) and Stanford Microarray Database.

### Microarray Analyses

Hierarchical clustering was performed with Cluster 3.0 [116] and visualized with Java TreeView 1.0.12 [117].

For SAM, unpaired two-class *t*-tests were performed with default settings (R-package samr; http://cran.r-project.org/web/packages/samr/index.html). Microarray features that passed quality filtering in all experiments were used as input. Ago IP experiments (Dataset S1) and mRNA expression experiments (Dataset S4) were mean centered at log_2_ 0 prior to running SAM. The ribosome occupancy (Dataset S2) and ribosome number/density measurements (Dataset S3) from miR-124 and mock-transfected cells were highly correlated, but had slightly different means (see main text). Because of the small changes in ribosome occupancy and ribosome density between miR-124–transfected and mock-transfected samples, we conservatively adjusted the means of each experiment to be the same by subtracting the difference between the mean of that experiment and the mean of all the experiments to ensure that differences observed between miR-124–transfected and mock-transfected cells were not due to the doping control normalization.

Enrichment of GO terms was performed with Genetrail [118]. *p*-Values were corrected for multiple-hypothesis testing by the Bonferroni method [119].

The significance of correlations was estimated in R by recalculating the correlations with 10,000 permuted sets of data, then estimating the *p*-value with the normal distribution function using the mean and standard deviation from the permuted data.

We used a bootstrap method to estimate 95% confidence intervals for the average changes in mRNA abundance, estimated translation rate, ribosome occupancy, and ribosome density (Figures 4 and 5, and Figure S8) of IP targets compared to nontargets. To do this, we sampled with replacement measurements for each gene from the mock and miR-124 replicates, respectively, 10,000 times, then calculated the respective changes between miR-124 IP targets and nontargets for the 10,000 bootstrapped samples.

### Sequence Data

For molecular features that mapped to genomic loci with an Entrez ID, the RefSeq sequence with the longest 3′-UTR was used. In cases with multiple RefSeqs with the same 3′-UTR length, the one that was alphanumerically first was used. RefSeq 3′-UTR, coding, and 5′-UTR sequences were retrieved from UCSC genome browser (hg18) http://genome.ucsc.edu/. Seed match sites in these sequences were identified with Perl scripts. miR-124 seed matches: 6mer_n2-7 “UGCCUU,” 6mer_n3-8 “GUGCCU,” 7mer-m8 “GUGCCUU,” 7mer-A1 “UGCCUUA,” 8mer “GUGCCUUA.” In many instances, there were multiple probes on the DNA microarrays that mapped to the same RefSeq. In these cases, we used the probe that was most enriched in Ago IPs from miR-124–transfected cells compared to mock-transfected cells.

## Supporting Information

Dataset S1 **Ago IP microarray data and SAM results.**
(4.54 MB XLS)

Dataset S2 **Ribosome occupancy values from miR-124 and mock-transfected cells and SAM results.**
(6.39 MB XLS)

Dataset S3 **Ribosome number values from miR-124 and mock-transfected cells and SAM results.**
(6.48 MB XLS)

Dataset S4 **mRNA expression microarray data and SAM results.**
(6.80 MB XLS)

Dataset S5 **Compendium of miR-124 data used for most analyses.**
(5.14 MB XLS)

Figure S1 **mRNA enrichment profiles in Ago IPs.** Unsupervised hierarchical clustering of the enrichment profiles in Ago IPs from miR-124-transfected cells (black), mock-transfected cells (blue), and negative control IPs (no Ago antibody) from both types of cells (orange). Rows correspond to 16,095 sequences (representing 9,729 genomic loci with a Refseq sequence), and columns represent individual experiments. Unsupervised hierarchical cluster analysis segregated the Ago IPs from the negative-control IPs. The Ago IPs were further bifurcated into two subgroups: miR-124 transfected and mock transfected. There was a significant correlation between Ago and mock IPs (*r* = 0.6), even though ∼10-fold less RNA was obtained from mock IPs compared to Ago IPs, and no protein bands were detectable by protein staining (unpublished data). We speculate that Ago complexes bind the beads nonspecifically and contribute significantly to the weak background binding (Text S1 and Figure S2).
(0.26 MB PDF)

Figure S2 **Streptavidin-coated Dynal beads weakly enrich miR-124 targets after miR-124 transfection.** (A) Supervised hierarchical clustering of the enrichment profiles of the 500 most enriched mRNAs in negative-control IPs from miR-124-transfected cells (blue) compared to mock-transfected cells (black). Rows correspond to mRNAs, and columns represent individual experiments. (B) Enrichment of seed matches to miR-124 in the 3′-UTRs of mRNAs nonspecifically associated with magnetic beads. The significance of enrichment of seed matches in Ago IP targets was measured with the hypergeometric distribution.
(0.31 MB PDF)

Figure S3 **Polysome profiles and doping control fits.** (A) Polysome profile traces from lysates prepared from mock-and miR-124 transfected HEK293T cells. The vertical gray line indicates the division between unbound and ribosome bound fractions. (B) Scatterplot between the observed “gradient-encoding” ratios of 85 exogenous RNAs doped into each of the ribosome bound fractions and their predicted ratios. The blue circles show the raw data and the blue line is the least-squares fit of the raw data, while the red circles and red line represent the corrected data and fit, respectively. (C) Scatterplot between the observed ratios of eight exogenous RNAs doped into each ribosome bound and ribosome unbound fractions and their predicted ratios. The blue circles show the raw data, and the blue line represents the least-squares fit of the raw data, while the red circles and red line represent the corrected data and fit, respectively.
(2.71 MB PDF)

Figure S4 **miR-124 Ago IP targets are likely destroyed, rather than deadenylated and stored.** Scatterplot between changes in mRNA abundance for miR-124 Ago IP targets following transfection with miR-124 compared to mock measured with poly(A) amplified mRNA (*x*-axis) and cDNA synthesized from randomly primed total RNA (*y*-axis). The red line has a slope of one and goes through the *y*-axis at zero. The black line is a least-squares fit of the data (slope = 0.82 in linear space, Pearson correlation log2 [mRNA] = 0.82). This analysis compares 208 miR-124 targets for which we obtained quality measurements in both experiments.
(0.34 MB PDF)

Figure S5 **Relationship between the coding sequence length and changes in ribosome occupancy and ribosome density of miR-124 Ago IP targets following transfection of miR-124.** (A) Scatterplot between coding sequence length (x-axis) and fold-change in ribosome occupancy (*y*-axis) for miR-124 Ago IP targets following transfection with miR-124 compared to mock. The horizontal gray line denotes a 20% reduction in ribosome occupancy. (B) Scatterplot between coding sequence length (*x*-axis) and fold-change in ribosome density (*y*-axis) for miR-124 Ago IP targets following transfection with miR-124 compared to mock. The red curve is a nonlinear least-squares fit of the change in ribosome density following a first-order decay as a function of coding sequence length.
(1.17 MB PDF)

Figure S6 **Relationship between ribosome occupancy in mock-transfected cells and change in ribosome occupancy following transfection of miR-124.** (A) Scatterplot between changes in ribosome occupancy (*x*-axis) and ribosome density (*y*-axis) for miR-124 Ago IP targets following transfection with miR-124 compared to mock. The gray line is a least-squares linear regression fit of the data (Spearman rank correlation = 0.45), and the red line is a moving average plot (window of 10). (B) The logarithm of the ratio of the average ribosome occupancy in miR-124-transfected cells to that in mock-transfected cells as a function of the average ribosome occupancy in mock-transfected cells (Spearman rank correlation = −0.78). Black circles correspond to mRNAs that were not enriched by the Ago IP following miR-124 transfection. Red circles correspond to mRNAs that were enriched by the Ago IP following miR-124 transfection (1% local FDR). The green curve represents a Lowess smoothed fit of the data. The gray curve shows the maximum possible increase in ribosome occupancy in miR-124 cells compared to mock cells. (C) The ratio in the average ribosome occupancy in miR-124-transfected cells versus mock-transfected cells minus the Lowess fit of the data (green points in (B)) as a function of the average ribosome occupancy in mock-transfected cells. (D) Scatterplot of changes in mRNA abundance (*x*-axis) versus changes in translation rate (*y*-axis) for Ago IP targets following transfection with miR-124. The slope of the least-squares fit of the data is 0.24 (in linear space, 0.36), and the Pearson correlation is 0.60. This is Figure 7 replotted to allow side-by-side comparison with (E). (E) Same as in (D), except that the changes in translation rate were obtained using the smoothed-fit adjusted ribosome occupancy measurements (C). The slope of the least-squares fit of the data is 0.20 (in linear space, 0.29), and the Pearson correlation is 0.59.
(3.06 MB PDF)

Figure S7 **Significance of the correlation between changes in mRNA abundance and translation of miR-124 Ago IP targets.** (A) Histogram of correlations between changes in mRNA abundance and translation for 100,000 permuted sets of miR-124 Ago IP targets. The red curve shows a normal distribution with mean (0.0) and standard deviation (.04) from the 100,000 permuted sets. The red arrow shows the Pearson correlation of the actual data (0.60, *p*<10^−45^). (B) Moving average plot of the Pearson correlation (window of 500) between changes in mRNA abundance and translation as a function of enrichment in Ago IPs in miR-124-transfected cells versus mock-transfected cells (SAM D-score). The horizontal grey line shows the average Pearson correlation between changes in mRNA abundance and translation across the moving windows (*r* = 0.09). (C) Histogram of correlations between changes in mRNA abundance and translation for 10,000 permuted sets of mRNAs that are not miR-124 targets, but have similar distribution in their changes in mRNA abundance (*t*-test, *p*>0.001) to miR-124 IP targets that change less than 40% in mRNA abundance. The red curve shows a normal distribution with mean (0.14) and standard deviation (0.04) from the 10,000 permuted sets. The red arrow shows the Pearson correlation of miR-124 IP targets that change less than 40% in mRNA abundance (*r* = 0.30, *p*<10^−5^).
(0.39 MB PDF)

Figure S8 **Concordant changes in mRNA abundance and translation of miR-124 Ago IP targets with 7mer 3′-UTR seed matches and miR-124 Ago IP targets that lack a 7mer 3′-UTR seed match.** (A) Scatterplot between changes in mRNA abundance (*x*-axis) and the estimated translation rate (*y*-axis) for miR-124 Ago IP targets with 7mer 3′-UTR seed matches following transfection with miR-124 compared to mock. The gray line is a least-squares linear regression fit of the data, and the black line is a moving average plot (window of 10). The slope of the least-squares fit of the data = 0.23 (in linear space = 0.37) and the Pearson correlation = 0.59. (B) Scatterplot between changes in mRNA abundance (*x*-axis) and the estimated translation rate (*y*-axis) for miR-124 Ago IP targets that lack 7mer 3′-UTR seed matches following transfection with miR-124 compared to mock. The gray line is a least-squares linear regression fit of the data, and the red line is a moving average plot (window of 10). The slope of the least-squares fit of the data = 0.21 (in linear space = 0.24) and the Pearson correlation = 0.42.
(0.35 MB PDF)

Figure S9 **Changes in abundance and translation of miR-124 Ago IP targets with seed matches in 3′-UTRs, coding sequences and 5′-UTRs.** (A) Cumulative distribution of the change in mRNA levels following transfection with miR-124 compared to mock. This analysis compares miR-124 Ago IP targets (1% local FDR) with at least one 3′-UTR 7mer seed match, but no coding sequence or 5′-UTR 7mer seed matches (red, 244), IP targets with at least one 3′-UTR 6mer seed match (green, 47), but no 3′-UTR, coding sequence, or 5′-UTR 7mer seed matches, IP targets with at least one coding sequence 7mer seed match, but no 3′-UTR or 5′-UTR 7mer seed matches (blue, 70), IP targets that lacked a 6mer seed match in the 3′-UTR, coding sequence, or 5′-UTR (orange,23), and nontargets (7385, black). This analysis compares Ago IP targets (red) versus nontargets (black). (B) Cumulative distribution of the change in translation following transfection with miR-124 compared to mock. This analysis compares miR-124 Ago IP targets (1% local FDR) with at least one 3′-UTR 7mer seed match, but no coding sequence or 5′-UTR 7mer seed matches (red), IP targets with at least one 3′-UTR 6mer seed match (green), but no 7mer seed matches in the 3′-UTR, coding sequence, or 5′-UTR, IP targets with at least one coding sequence 7mer seed match, but no 7mer seed match in the 3′-UTR or 5′-UTR (blue), IP targets that lacked a 6mer seed match in the 3′-UTR, coding sequence, or 5′-UTR (orange), and nontargets (black). This analysis compares Ago IP targets (red) versus nontargets (black). (C) Bar plot of the average change in mRNA abundance (blue) and translation rate (red) of miR-124 Ago IP targets following transfection with miR-124. The average change in mRNA abundance and translation of targets was calculated by subtracting the average change of nontargets for the mRNA abundance and translation rate measurements following transfection with miR-124. This analysis compares miR-124 Ago IP targets (1% local FDR) with at least one 3′-UTR 7mer seed match, but no coding sequence or 5′-UTR 7mer seed matches, IP targets with at least one 3′-UTR 6mer seed match, but no 7mer seed matches in the 3′-UTR, coding sequence, or 5′-UTR, IP targets with at least one coding sequence 7mer seed match, but no 7mer seed match in the 3′-UTR or 5′-UTR, IP targets with at least one 7mer seed match in the 5′-UTR, but no 7mer seed match in the 3′-UTR or coding sequence, and IP targets that lacked a 6mer seed match in the 3′-UTR, coding sequence, or 5′-UTR. This analysis compares Ago IP targets (red) versus nontargets (black). The error bars represent 95% confidence intervals in the mean difference estimated by bootstrap analysis.
(0.65 MB PDF)

Figure S10 **Efficiency of recruitment to Argonautes by miR-124 seed matches correlates with effects on both mRNA abundance and translation.** (A) Scatterplot between changes in Ago IP enrichment (*x*-axis) following transfection with miR-124 compared to mock and estimated changes in protein production (Equation 2) (*y*-axis) for mRNAs with either 8mer seed matches (red dots) or 7mer seed matches (blue dots) to miR-124 in their 3′-UTRs. For 8mer seed matches, the slope of the least-squares fit of the log_2_ data is −0.46 (in linear space, −0.03), and the log_2_ Pearson correlation is −0.72. For 7mer seed matches, the slope of the least-squares fit of the log_2_ data is −0.39 (in linear space, −0.05), and the log2 Pearson correlation is −0.72. (B) Same as (A) except for mRNAs with seed matches to miR-124 in their coding sequences and no 7mer seed matches in their 3′-UTRs. For 8mer seed matches, the slope of the least-squares fit of the log_2_ data is −0.13 (in linear space, −0.008), and the log2 Pearson correlation is −0.39. For 7mer seed matches, the slope of the least-squares fit of the log_2_ data = −0.14 (in linear space, −0.02), and the log_2_ Pearson correlation is 0.38. (C) Same as in (A) except for mRNAs with 6mer seed matches to miR-124 in their 3′-UTR, but no 7mer seed match in their 3′-UTR or coding sequence (red), and mRNAs that lack 6mer seed matches in their 3′-UTR or coding sequence (blue). For 6mer seed matches, the slope of the least-squares fit of the log_2_ data is −0.21 (in linear space, −0.09), and the log_2_ Pearson correlation is −0.40. For mRNAs without 6mer seed matches, the slope of the least-squares fit of the log_2_ data is −0.13 (in linear space, −0.07), and the log_2_ Pearson correlation is −0.22.
(1.71 MB PDF)

Table S1 **Summary of miR-124 targets for Western blot analysis.**
(0.02 MB PDF)

Table S2 **Exogenous doping control information.**
(0.05 MB XLS)

Text S1 **miRNA-effector complexes appear to nonspecifically bind streptavidin-coated Dynal beads.**
(0.03 MB DOC)

Text S2 **Enrichment of seed matches to highly expressed miRNAs in Ago IPs from mock-transfected cells.**
(0.03 MB DOC)

Text S3 **Relationship between ribosome occupancy in mock-transfected cells and changes in ribosome occupancy following transfection of miR-124.**
(0.03 MB DOC)

Text S4 **Evaluation of the significance of the correlation between changes in mRNA abundance and translation of miR-124 Ago IP targets following transfection with miR-124.**
(0.05 MB DOC)

## Abbreviations

FDR: false-discovery rate
IP: immunopurification
miRNA: microRNA
nts: nucleotides
SAM: significance analysis of microarrays

## References

[1] Lee R. C, Feinbaum R. L, Ambros V (1993). The C. elegans heterochronic gene lin-4 encodes small RNAs with antisense complementarity to lin-14.. Cell.

[2] Bartel D. P (2004). MicroRNAs: genomics, biogenesis, mechanism, and function.. Cell.

[3] Filipowicz W, Bhattacharyya S. N, Sonenberg N (2008). Mechanisms of post-transcriptional regulation by microRNAs: are the answers in sight?. Nat Rev Genet.

[4] Lewis B. P, Burge C. B, Bartel D. P (2005). Conserved seed pairing, often flanked by adenosines, indicates that thousands of human genes are microRNA targets.. Cell.

[5] Friedman R. C, Farh K. K, Burge C. B, Bartel D (2009). Most mammalian mRNAs are conserved targets of microRNAs.. Genome Res.

[6] Grimson A, Srivastava M, Fahey B, Woodcroft B. J, Chiang H. R (2008). Early origins and evolution of microRNAs and Piwi-interacting RNAs in animals.. Nature.

[7] Olsen P. H, Ambros V (1999). The lin-4 regulatory RNA controls developmental timing in Caenorhabditis elegans by blocking LIN-14 protein synthesis after the initiation of translation.. Dev Biol.

[8] Ambros V (2004). The functions of animal microRNAs.. Nature.

[9] Bagga S, Bracht J, Hunter S, Massirer K, Holtz J (2005). Regulation by let-7 and lin-4 miRNAs results in target mRNA degradation.. Cell.

[10] Humphreys D. T, Westman B. J, Martin D. I, Preiss T (2005). MicroRNAs control translation initiation by inhibiting eukaryotic initiation factor 4E/cap and poly(A) tail function.. Proc Natl Acad Sci U S A.

[11] Lim L. P, Lau N. C, Garrett-Engele P, Grimson A, Schelter J. M (2005). Microarray analysis shows that some microRNAs downregulate large numbers of target mRNAs.. Nature.

[12] Wu L, Belasco J. G (2005). Micro-RNA regulation of the mammalian lin-28 gene during neuronal differentiation of embryonal carcinoma cells.. Mol Cell Biol.

[13] Valencia-Sanchez M. A, Liu J, Hannon G. J, Parker R (2006). Control of translation and mRNA degradation by miRNAs and siRNAs.. Genes Dev.

[14] Wu L, Fan J, Belasco J. G (2006). MicroRNAs direct rapid deadenylation of mRNA.. Proc Natl Acad Sci U S A.

[15] Farh K. K, Grimson A, Jan C, Lewis B. P, Johnston W. K (2005). The widespread impact of mammalian MicroRNAs on mRNA repression and evolution.. Science.

[16] Baek D, Villen J, Shin C, Camargo F. D, Gygi S. P (2008). The impact of microRNAs on protein output.. Nature.

[17] Selbach M, Schwanhausser B, Thierfelder N, Fang Z, Khanin R (2008). Widespread changes in protein synthesis induced by microRNAs.. Nature.

[18] Abbott A. L, Alvarez-Saavedra E, Miska E. A, Lau N. C, Bartel D. P (2005). The let-7 MicroRNA family members mir-48, mir-84, and mir-241 function together to regulate developmental timing in Caenorhabditis elegans.. Dev Cell.

[19] Alvarez-Garcia I, Miska E. A (2005). MicroRNA functions in animal development and human disease.. Development.

[20] Hatfield S. D, Shcherbata H. R, Fischer K. A, Nakahara K, Carthew R. W (2005). Stem cell division is regulated by the microRNA pathway.. Nature.

[21] Kloosterman W. P, Plasterk R. H (2006). The diverse functions of microRNAs in animal development and disease.. Dev Cell.

[22] Linsley P. S, Schelter J, Burchard J, Kibukawa M, Martin M. M (2007). Transcripts targeted by the microRNA-16 family cooperatively regulate cell cycle progression.. Mol Cell Biol.

[23] Lu J, Getz G, Miska E. A, Alvarez-Saavedra E, Lamb J (2005). MicroRNA expression profiles classify human cancers.. Nature.

[24] Reinhart B. J, Slack F. J, Basson M, Pasquinelli A. E, Bettinger J. C (2000). The 21-nucleotide let-7 RNA regulates developmental timing in Caenorhabditis elegans.. Nature.

[25] Jackson R. J, Standart N (2007). How do microRNAs regulate gene expression?. Sci STKE.

[26] Kozak M (2008). Faulty old ideas about translational regulation paved the way for current confusion about how microRNAs function.. Gene.

[27] Nissan T, Parker R (2008). Computational analysis of miRNA-mediated repression of translation: implications for models of translation initiation inhibition.. RNA.

[28] Shyu A. B, Wilkinson M. F, van Hoof A (2008). Messenger RNA regulation: to translate or to degrade.. EMBO J.

[29] Wu L, Belasco J. G (2008). Let me count the ways: mechanisms of gene regulation by miRNAs and siRNAs.. Mol Cell.

[30] Seggerson K, Tang L, Moss E. G (2002). Two genetic circuits repress the Caenorhabditis elegans heterochronic gene lin-28 after translation initiation.. Dev Biol.

[31] Lee R. C, Ambros V (2001). An extensive class of small RNAs in Caenorhabditis elegans.. Science.

[32] Slack F, Ruvkun G (1997). Temporal pattern formation by heterochronic genes.. Annu Rev Genet.

[33] Wightman B, Ha I, Ruvkun G (1993). Posttranscriptional regulation of the heterochronic gene lin-14 by lin-4 mediates temporal pattern formation in C. elegans.. Cell.

[34] Zeng Y, Cullen B. R (2003). Sequence requirements for micro RNA processing and function in human cells.. RNA.

[35] Zeng Y, Wagner E. J, Cullen B. R (2002). Both natural and designed micro RNAs can inhibit the expression of cognate mRNAs when expressed in human cells.. Mol Cell.

[36] Doench J. G, Petersen C. P, Sharp P. A (2003). siRNAs can function as miRNAs.. Genes Dev.

[37] Doench J. G, Sharp P. A (2004). Specificity of microRNA target selection in translational repression.. Genes Dev.

[38] Bhattacharyya S. N, Habermacher R, Martine U, Closs E. I, Filipowicz W (2006). Relief of microRNA-mediated translational repression in human cells subjected to stress.. Cell.

[39] Chu C. Y, Rana T. M (2006). Translation repression in human cells by microRNA-induced gene silencing requires RCK/p54.. PLoS Biol.

[40] Ding L, Spencer A, Morita K, Han M (2005). The developmental timing regulator AIN-1 interacts with miRISCs and may target the argonaute protein ALG-1 to cytoplasmic P bodies in C. elegans.. Mol Cell.

[41] Liu J, Rivas F. V, Wohlschlegel J, Yates J. R, Parker R (2005). A role for the P-body component GW182 in microRNA function.. Nat Cell Biol.

[42] Liu J, Valencia-Sanchez M. A, Hannon G. J, Parker R (2005). MicroRNA-dependent localization of targeted mRNAs to mammalian P-bodies.. Nat Cell Biol.

[43] Rehwinkel J, Behm-Ansmant I, Gatfield D, Izaurralde E (2005). A crucial role for GW182 and the DCP1:DCP2 decapping complex in miRNA-mediated gene silencing.. RNA.

[44] Pillai R. S, Bhattacharyya S. N, Artus C. G, Zoller T, Cougot N (2005). Inhibition of translational initiation by Let-7 MicroRNA in human cells.. Science.

[45] Giraldez A. J, Mishima Y, Rihel J, Grocock R. J, Van Dongen S (2006). Zebrafish MiR-430 promotes deadenylation and clearance of maternal mRNAs.. Science.

[46] Bachvarova R. F (1992). A maternal tail of poly(A): the long and the short of it.. Cell.

[47] Deshpande A. K, Chatterjee B, Roy A. K (1979). Translation and stability of rat liver messenger RNA for alpha 2 mu-globulin in Xenopus oocyte. The role of terminal poly(A).. J Biol Chem.

[48] Drummond D. R, Armstrong J, Colman A (1985). The effect of capping and polyadenylation on the stability, movement and translation of synthetic messenger RNAs in Xenopus oocytes.. Nucleic Acids Res.

[49] Gallie D. R (1991). The cap and poly(A) tail function synergistically to regulate mRNA translational efficiency.. Genes Dev.

[50] Gallie D. R, Lucas W. J, Walbot V (1989). Visualizing mRNA expression in plant protoplasts: factors influencing efficient mRNA uptake and translation.. Plant Cell.

[51] Standart N, Jackson R. J (2007). MicroRNAs repress translation of m7Gppp-capped target mRNAs in vitro by inhibiting initiation and promoting deadenylation.. Genes Dev.

[52] Wakiyama M, Takimoto K, Ohara O, Yokoyama S (2007). Let-7 microRNA-mediated mRNA deadenylation and translational repression in a mammalian cell-free system.. Genes Dev.

[53] Eulalio A, Huntzinger E, Nishihara T, Rehwinkel J, Fauser M (2009). Deadenylation is a widespread effect of miRNA regulation.. RNA.

[54] Kiriakidou M, Tan G. S, Lamprinaki S, De Planell-Saguer M, Nelson P. T (2007). An mRNA m7G cap binding-like motif within human Ago2 represses translation.. Cell.

[55] Mathonnet G, Fabian M. R, Svitkin Y. V, Parsyan A, Huck L (2007). MicroRNA inhibition of translation initiation in vitro by targeting the cap-binding complex eIF4F.. Science.

[56] Thermann R, Hentze M. W (2007). Drosophila miR2 induces pseudo-polysomes and inhibits translation initiation.. Nature.

[57] Wang B, Love T. M, Call M. E, Doench J. G, Novina C. D (2006). Recapitulation of short RNA-directed translational gene silencing in vitro.. Mol Cell.

[58] Wang B, Yanez A, Novina C. D (2008). MicroRNA-repressed mRNAs contain 40S but not 60S components.. Proc Natl Acad Sci U S A.

[59] Lytle J. R, Yario T. A, Steitz J. A (2007). Target mRNAs are repressed as efficiently by microRNA-binding sites in the 5′ UTR as in the 3′ UTR.. Proc Natl Acad Sci U S A.

[60] Nottrott S, Simard M. J, Richter J. D (2006). Human let-7a miRNA blocks protein production on actively translating polyribosomes.. Nat Struct Mol Biol.

[61] Petersen C. P, Bordeleau M. E, Pelletier J, Sharp P. A (2006). Short RNAs repress translation after initiation in mammalian cells.. Mol Cell.

[62] Maroney P. A, Yu Y, Fisher J, Nilsen T. W (2006). Evidence that microRNAs are associated with translating messenger RNAs in human cells.. Nat Struct Mol Biol.

[63] Schmitter D, Filkowski J, Sewer A, Pillai R. S, Oakeley E. J (2006). Effects of Dicer and Argonaute down-regulation on mRNA levels in human HEK293 cells.. Nucleic Acids Res.

[64] Ding X. C, Grosshans H (2009). Repression of C. elegans microRNA targets at the initiation level of translation requires GW182 proteins.. EMBO J.

[65] Eulalio A, Rehwinkel J, Stricker M, Huntzinger E, Yang S. F (2007). Target-specific requirements for enhancers of decapping in miRNA-mediated gene silencing.. Genes Dev.

[66] Wu L, Fan J, Belasco J. G (2008). Importance of translation and nonnucleolytic ago proteins for on-target RNA interference.. Curr Biol.

[67] Aleman L. M, Doench J, Sharp P. A (2007). Comparison of siRNA-induced off-target RNA and protein effects.. RNA.

[68] Nilsen T. W (2007). Mechanisms of microRNA-mediated gene regulation in animal cells.. Trends Genet.

[69] Kong Y. W, Cannell I. G, de Moor C. H, Hill K, Garside P. G (2008). The mechanism of micro-RNA-mediated translation repression is determined by the promoter of the target gene.. Proc Natl Acad Sci U S A.

[70] Hendrickson D. G, Hogan D. J, Herschlag D, Ferrell J. E, Brown P. O (2008). Systematic identification of mRNAs recruited to argonaute 2 by specific microRNAs and corresponding changes in transcript abundance.. PLoS ONE.

[71] Easow G, Teleman A. A, Cohen S. M (2007). Isolation of microRNA targets by miRNP immunopurification.. RNA.

[72] Karginov F. V, Conaco C, Xuan Z, Schmidt B. H, Parker J. S (2007). A biochemical approach to identifying microRNA targets.. Proc Natl Acad Sci U S A.

[73] Ikeda K, Satoh M, Pauley K. M, Fritzler M. J, Reeves W. H (2006). Detection of the argonaute protein Ago2 and microRNAs in the RNA induced silencing complex (RISC) using a monoclonal antibody.. J Immunol Methods.

[74] Brennecke J, Stark A, Russell R. B, Cohen S. M (2005). Principles of microRNA-target recognition.. PLoS Biol.

[75] Krek A, Grun D, Poy M. N, Wolf R, Rosenberg L (2005). Combinatorial microRNA target predictions.. Nat Genet.

[76] Lewis B. P, Shih I. H, Jones-Rhoades M. W, Bartel D. P, Burge C. B (2003). Prediction of mammalian microRNA targets.. Cell.

[77] Xie X, Lu J, Kulbokas E. J, Golub T. R, Mootha V (2005). Systematic discovery of regulatory motifs in human promoters and 3′ UTRs by comparison of several mammals.. Nature.

[78] Forman J. J, Legesse-Miller A, Coller H. A (2008). A search for conserved sequences in coding regions reveals that the let-7 microRNA targets Dicer within its coding sequence.. Proc Natl Acad Sci U S A.

[79] Tay Y, Zhang J, Thomson A. M, Lim B, Rigoutsos I (2008). MicroRNAs to Nanog, Oct4 and Sox2 coding regions modulate embryonic stem cell differentiation.. Nature.

[80] Arava Y, Wang Y, Storey J. D, Liu C. L, Brown P. O (2003). Genome-wide analysis of mRNA translation profiles in Saccharomyces cerevisiae.. Proc Natl Acad Sci U S A.

[81] Wolin S. L, Walter P (1988). Ribosome pausing and stacking during translation of a eukaryotic mRNA.. EMBO J.

[82] Arava Y, Boas F. E, Brown P. O, Herschlag D (2005). Dissecting eukaryotic translation and its control by ribosome density mapping.. Nucleic Acids Res.

[83] Ingolia N. T, Ghaemmaghami S, Newman J. R, Weissman J. S (2009). Genome-wide analysis in vivo of translation with nucleotide resolution using ribosome profiling.. Science.

[84] Richter J. D (1999). Cytoplasmic polyadenylation in development and beyond.. Microbiol Mol Biol Rev.

[85] Wormington M (1993). Poly(A) and translation: development control.. Curr Opin Cell Biol.

[86] Wormington M (1994). Unmasking the role of the 3′ UTR in the cytoplasmic polyadenylation and translational regulation of maternal mRNAs.. Bioessays.

[87] Hegde R. S, Kang S. W (2008). The concept of translocational regulation.. J Cell Biol.

[88] Fang S, Jensen J. P, Ludwig R. L, Vousden K. H, Weissman A. M (2000). Mdm2 is a RING finger-dependent ubiquitin protein ligase for itself and p53.. J Biol Chem.

[89] Ito K, Adachi S, Iwakami R, Yasuda H, Muto Y (2001). N-Terminally extended human ubiquitin-conjugating enzymes (E2s) mediate the ubiquitination of RING-finger proteins, ARA54 and RNF8.. Eur J Biochem.

[90] Yang Y, Fang S, Jensen J. P, Weissman A. M, Ashwell J. D (2000). Ubiquitin protein ligase activity of IAPs and their degradation in proteasomes in response to apoptotic stimuli.. Science.

[91] Zeng Y, Yi R, Cullen B. R (2003). MicroRNAs and small interfering RNAs can inhibit mRNA expression by similar mechanisms.. Proc Natl Acad Sci U S A.

[92] Behm-Ansmant I, Rehwinkel J, Doerks T, Stark A, Bork P (2006). mRNA degradation by miRNAs and GW182 requires both CCR4:NOT deadenylase and DCP1:DCP2 decapping complexes.. Genes Dev.

[93] Grimson A, Farh K. K, Johnston W. K, Garrett-Engele P, Lim L. P (2007). MicroRNA targeting specificity in mammals: determinants beyond seed pairing.. Mol Cell.

[94] Marson A, Levine S. S, Cole M. F, Frampton G. M, Brambrink T (2008). Connecting microRNA genes to the core transcriptional regulatory circuitry of embryonic stem cells.. Cell.

[95] Stark A, Lin M. F, Kheradpour P, Pedersen J. S, Parts L (2007). Discovery of functional elements in 12 Drosophila genomes using evolutionary signatures.. Nature.

[96] Landthaler M, Yalcin A, Tuschl T (2004). The human DiGeorge syndrome critical region gene 8 and Its D. melanogaster homolog are required for miRNA biogenesis.. Curr Biol.

[97] Beitzinger M, Peters L, Zhu J. Y, Kremmer E, Meister G (2007). Identification of human microRNA targets from isolated argonaute protein complexes.. RNA Biol.

[98] Enright A. J, John B, Gaul U, Tuschl T, Sander C (2003). MicroRNA targets in Drosophila.. Genome Biol.

[99] Holtz J, Pasquinelli A. E (2009). Uncoupling of lin-14 mRNA and protein repression by nutrient deprivation in Caenorhabditis elegans.. RNA.

[100] Coller J, Parker R (2005). General translational repression by activators of mRNA decapping.. Cell.

[101] Kawai T, Fan J, Mazan-Mamczarz K, Gorospe M (2004). Global mRNA stabilization preferentially linked to translational repression during the endoplasmic reticulum stress response.. Mol Cell Biol.

[102] Sheth U, Parker R (2003). Decapping and decay of messenger RNA occur in cytoplasmic processing bodies.. Science.

[103] Schwartz D. C, Parker R (1999). Mutations in translation initiation factors lead to increased rates of deadenylation and decapping of mRNAs in Saccharomyces cerevisiae.. Mol Cell Biol.

[104] Bandyopadhyay R, Coutts M, Krowczynska A, Brawerman G (1990). Nuclease activity associated with mammalian mRNA in its native state: possible basis for selectivity in mRNA decay.. Mol Cell Biol.

[105] Caruccio N, Ross J (1994). Purification of a human polyribosome-associated 3′ to 5′ exoribonuclease.. J Biol Chem.

[106] Grafi G, Sela I, Galili G (1993). Translational regulation of human beta interferon mRNA: association of the 3′ AU-rich sequence with the poly(A) tail reduces translation efficiency in vitro.. Mol Cell Biol.

[107] Jacobson A, Peltz S. W (1996). Interrelationships of the pathways of mRNA decay and translation in eukaryotic cells.. Annu Rev Biochem.

[108] Kruys V, Marinx O, Shaw G, Deschamps J, Huez G (1989). Translational blockade imposed by cytokine-derived UA-rich sequences.. Science.

[109] Kruys V, Wathelet M, Poupart P, Contreras R, Fiers W (1987). The 3′ untranslated region of the human interferon-beta mRNA has an inhibitory effect on translation.. Proc Natl Acad Sci U S A.

[110] Kruys V. I, Wathelet M. G, Huez G. A (1988). Identification of a translation inhibitory element (TIE) in the 3′ untranslated region of the human interferon-beta mRNA.. Gene.

[111] Marinx O, Bertrand S, Karsenti E, Huez G, Kruys V (1994). Fertilization of Xenopus eggs imposes a complete translational arrest of mRNAs containing 3′UUAUUUAU elements.. FEBS Lett.

[112] Muhlrad D, Decker C. J, Parker R (1995). Turnover mechanisms of the stable yeast PGK1 mRNA.. Mol Cell Biol.

[113] Klapholz-Brown Z, Walmsley G. G, Nusse Y. M, Nusse R, Brown P. O (2007). Transcriptional program induced by Wnt protein in human fibroblasts suggests mechanisms for cell cooperativity in defining tissue microenvironments.. PLoS ONE.

[114] Fare T. L, Coffey E. M, Dai H, He Y. D, Kessler D. A (2003). Effects of atmospheric ozone on microarray data quality.. Anal Chem.

[115] Demeter J, Beauheim C, Gollub J, Hernandez-Boussard T, Jin H (2007). The Stanford Microarray Database: implementation of new analysis tools and open source release of software.. Nucleic Acids Res.

[116] Eisen M. B, Spellman P. T, Brown P. O, Botstein D (1998). Cluster analysis and display of genome-wide expression patterns.. Proc Natl Acad Sci U S A.

[117] Saldanha A. J (2004). Java Treeview: extensible visualization of microarray data.. Bioinformatics.

[118] Backes C, Keller A, Kuentzer J, Kneissl B, Comtesse N (2007). GeneTrail: advanced gene set enrichment analysis.. Nucleic Acids Res.

[119] Holm S (1979). A simple sequentially rejective multiple test procedure.. Scand J Statist.

