# Supplementary figures and images for: Concordant Regulation of Translation and mRNA Abundance for Hundreds of Targets of a Human microRNA
Source: PLoS Biol. 2009 Nov 10;7(11):e1000238. doi: 10.1371/journal.pbio.1000238 (PMC2766070; doi:10.1371/journal.pbio.1000238)

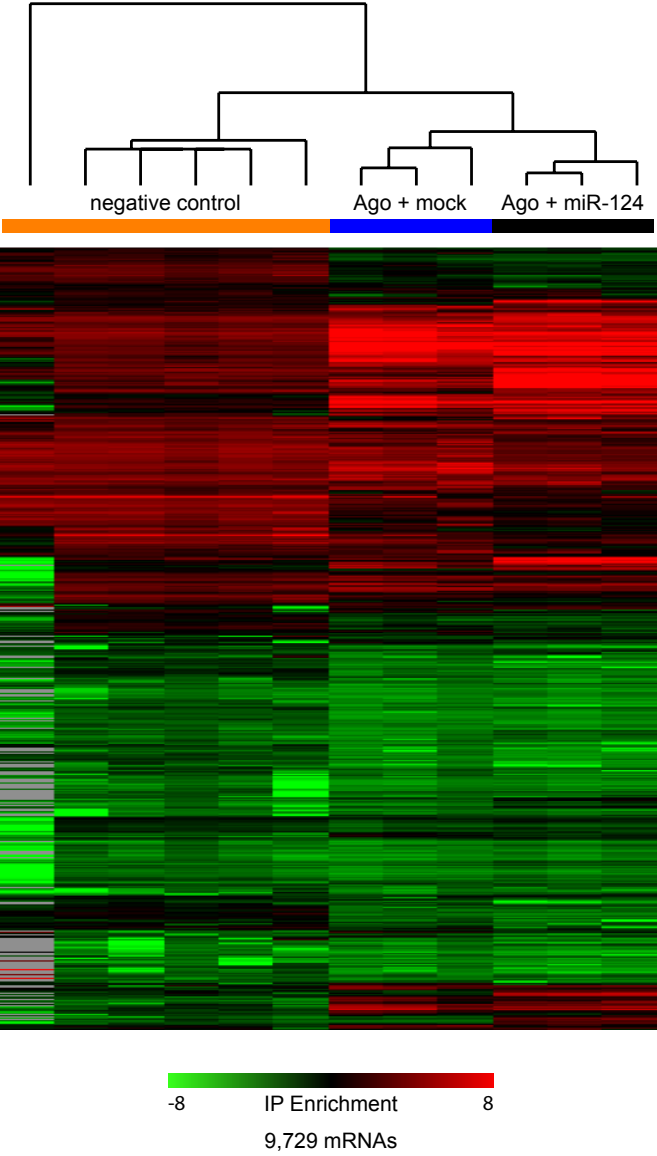

Supplement: Figure S1 — mRNA enrichment profiles in Ago IPs. Unsupervised hierarchical clustering of the enrichment profiles in Ago IPs from miR-124-transfected cells (black), mock-transfected cells (blue), and negative control IPs (no Ago antibody) from both types of cells (orange). Rows correspond to 16,095 sequences (representing 9,729 genomic loci with a Refseq sequence), and columns represent individual experiments. Unsupervised hierarchical cluster analysis segregated the Ago IPs from the negative-control IPs. The Ago IPs were further bifurcated into two subgroups: miR-124 transfected and mock transfected. There was a significant correlation between Ago and mock IPs (r = 0.6), even though ∼10-fold less RNA was obtained from mock IPs compared to Ago IPs, and no protein bands were detectable by protein staining (unpublished data). We speculate that Ago complexes bind the beads nonspecifically and contribute significantly to the weak background binding (Text S1 and Figure S2). (0.26 MB PDF) [file pbio.1000238.s006.pdf]

A

Mock 1

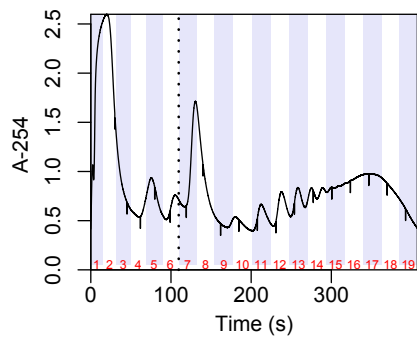

B

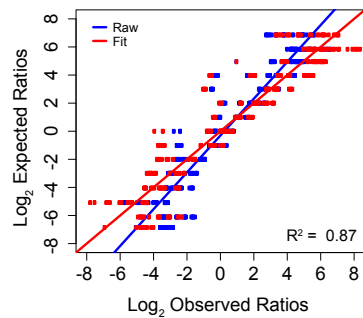

C

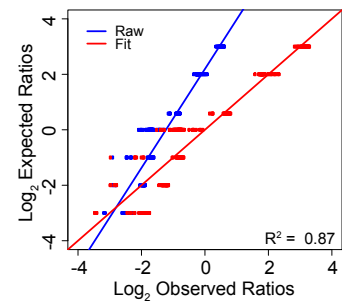

Mock 2

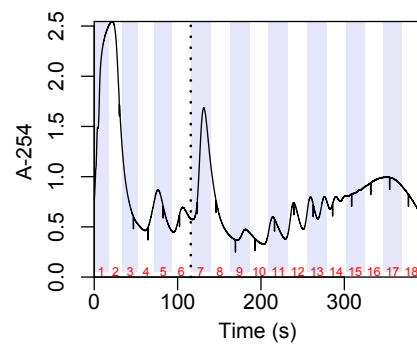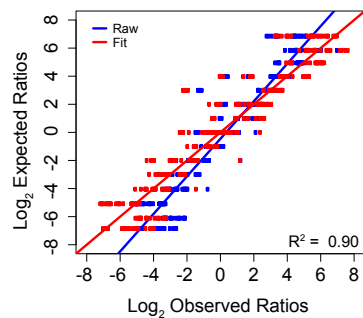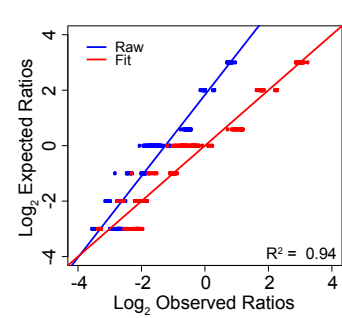

Mock 3

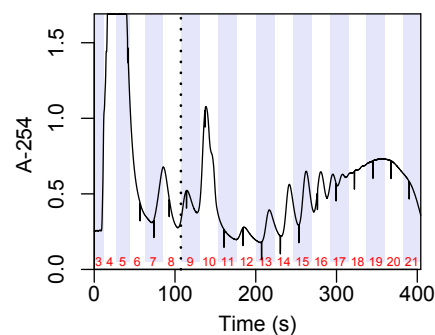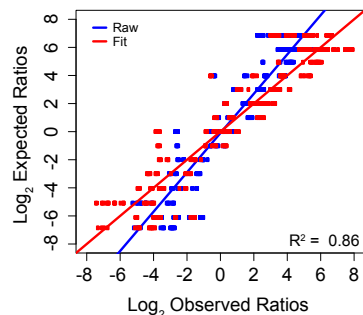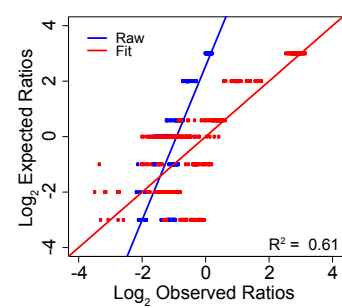

miR-124 1

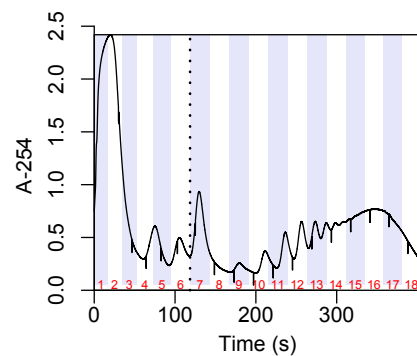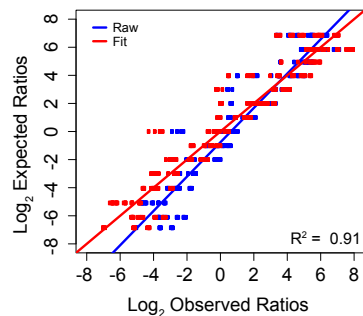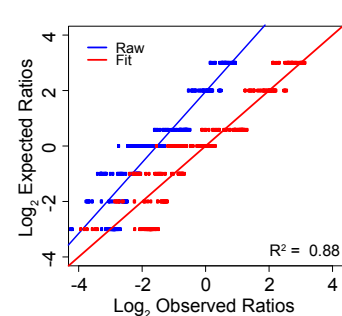

miR-124 2

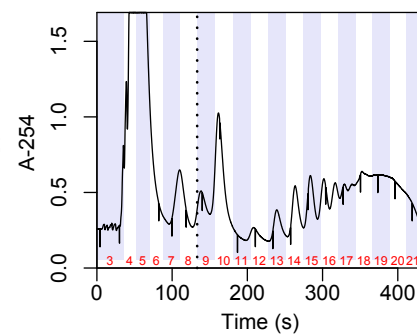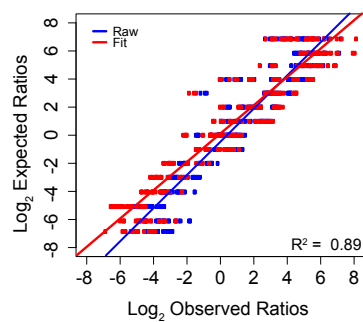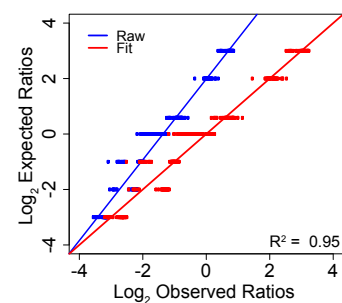

Supplement: Figure S3 — Polysome profiles and doping control fits. (A) Polysome profile traces from lysates prepared from mock-and miR-124 transfected HEK293T cells. The vertical gray line indicates the division between unbound and ribosome bound fractions. (B) Scatterplot between the observed “gradient-encoding” ratios of 85 exogenous RNAs doped into each of the ribosome bound fractions and their predicted ratios. The blue circles show the raw data and the blue line is the least-squares fit of the raw data, while the red circles and red line represent the corrected data and fit, respectively. (C) Scatterplot between the observed ratios of eight exogenous RNAs doped into each ribosome bound and ribosome unbound fractions and their predicted ratios. The blue circles show the raw data, and the blue line represents the least-squares fit of the raw data, while the red circles and red line represent the corrected data and fit, respectively. (2.71 MB PDF) [file pbio.1000238.s008.pdf]

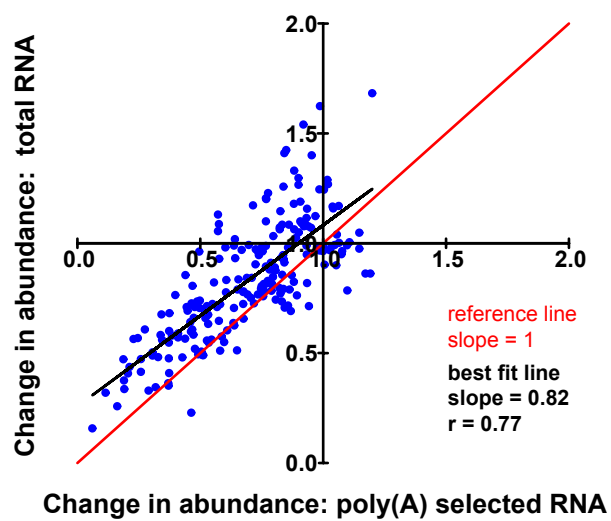

Supplement: Figure S4 — miR-124 Ago IP targets are likely destroyed, rather than deadenylated and stored. Scatterplot between changes in mRNA abundance for miR-124 Ago IP targets following transfection with miR-124 compared to mock measured with poly(A) amplified mRNA (x-axis) and cDNA synthesized from randomly primed total RNA (y-axis). The red line has a slope of one and goes through the y-axis at zero. The black line is a least-squares fit of the data (slope = 0.82 in linear space, Pearson correlation log2 [mRNA] = 0.82). This analysis compares 208 miR-124 targets for which we obtained quality measurements in both experiments. (0.34 MB PDF) [file pbio.1000238.s009.pdf]

A

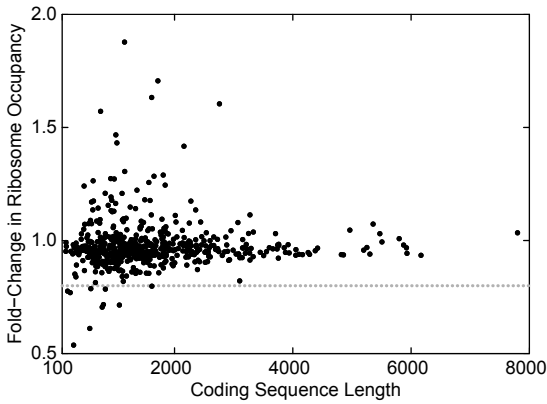

B

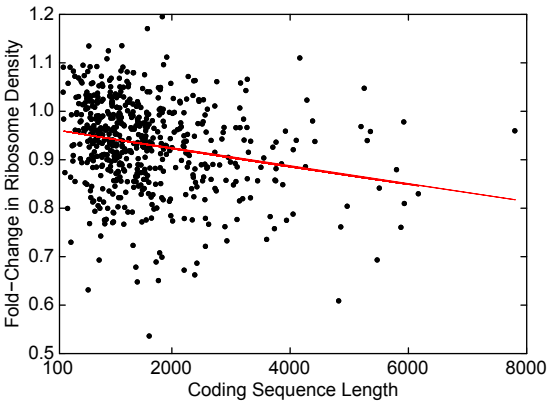

Supplement: Figure S5 — Relationship between the coding sequence length and changes in ribosome occupancy and ribosome density of miR-124 Ago IP targets following transfection of miR-124. (A) Scatterplot between coding sequence length (x-axis) and fold-change in ribosome occupancy (y-axis) for miR-124 Ago IP targets following transfection with miR-124 compared to mock. The horizontal gray line denotes a 20% reduction in ribosome occupancy. (B) Scatterplot between coding sequence length (x-axis) and fold-change in ribosome density (y-axis) for miR-124 Ago IP targets following transfection with miR-124 compared to mock. The red curve is a nonlinear least-squares fit of the change in ribosome density following a first-order decay as a function of coding sequence length. (1.17 MB PDF) [file pbio.1000238.s010.pdf]

A

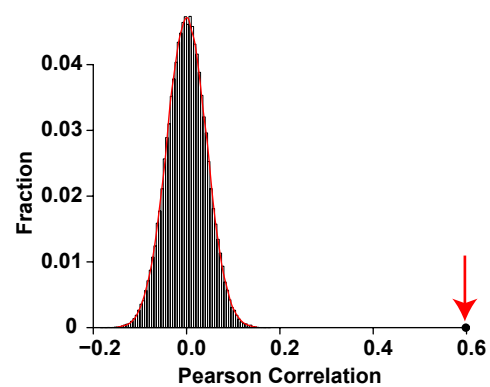

B

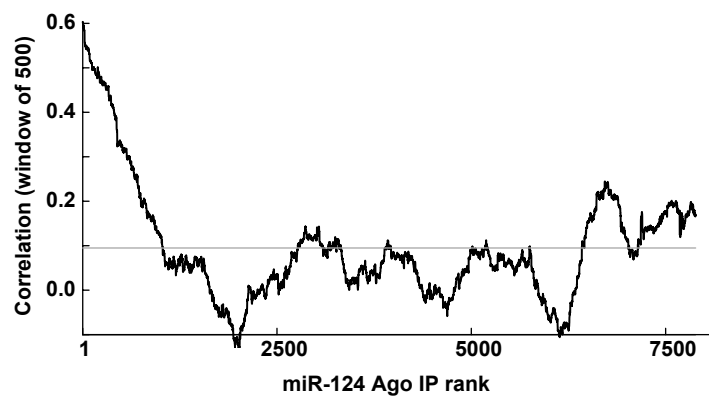

C

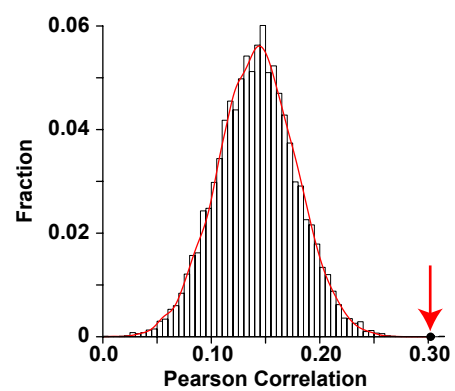

Supplement: Figure S7 — Significance of the correlation between changes in mRNA abundance and translation of miR-124 Ago IP targets. (A) Histogram of correlations between changes in mRNA abundance and translation for 100,000 permuted sets of miR-124 Ago IP targets. The red curve shows a normal distribution with mean (0.0) and standard deviation (.04) from the 100,000 permuted sets. The red arrow shows the Pearson correlation of the actual data (0.60, p<10−45). (B) Moving average plot of the Pearson correlation (window of 500) between changes in mRNA abundance and translation as a function of enrichment in Ago IPs in miR-124-transfected cells versus mock-transfected cells (SAM D-score). The horizontal grey line shows the average Pearson correlation between changes in mRNA abundance and translation across the moving windows (r = 0.09). (C) Histogram of correlations between changes in mRNA abundance and translation for 10,000 permuted sets of mRNAs that are not miR-124 targets, but have similar distribution in their changes in mRNA abundance (t-test, p>0.001) to miR-124 IP targets that change less than 40% in mRNA abundance. The red curve shows a normal distribution with mean (0.14) and standard deviation (0.04) from the 10,000 permuted sets. The red arrow shows the Pearson correlation of miR-124 IP targets that change less than 40% in mRNA abundance (r = 0.30, p<10−5). (0.39 MB PDF) [file pbio.1000238.s012.pdf]

A

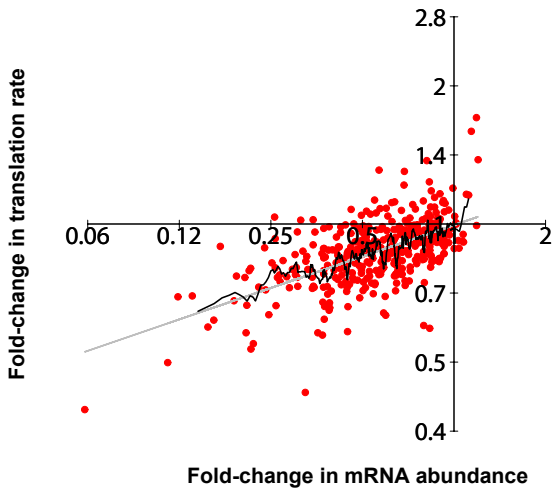

B

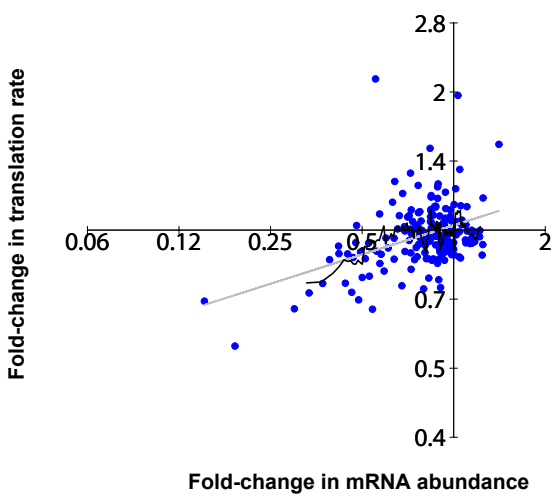

Supplement: Figure S8 — Concordant changes in mRNA abundance and translation of miR-124 Ago IP targets with 7mer 3′-UTR seed matches and miR-124 Ago IP targets that lack a 7mer 3′-UTR seed match. (A) Scatterplot between changes in mRNA abundance (x-axis) and the estimated translation rate (y-axis) for miR-124 Ago IP targets with 7mer 3′-UTR seed matches following transfection with miR-124 compared to mock. The gray line is a least-squares linear regression fit of the data, and the black line is a moving average plot (window of 10). The slope of the least-squares fit of the data = 0.23 (in linear space = 0.37) and the Pearson correlation = 0.59. (B) Scatterplot between changes in mRNA abundance (x-axis) and the estimated translation rate (y-axis) for miR-124 Ago IP targets that lack 7mer 3′-UTR seed matches following transfection with miR-124 compared to mock. The gray line is a least-squares linear regression fit of the data, and the red line is a moving average plot (window of 10). The slope of the least-squares fit of the data = 0.21 (in linear space = 0.24) and the Pearson correlation = 0.42. (0.35 MB PDF) [file pbio.1000238.s013.pdf]

A

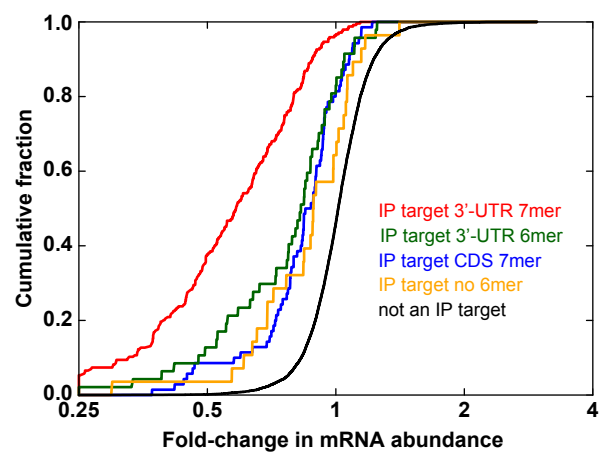

B

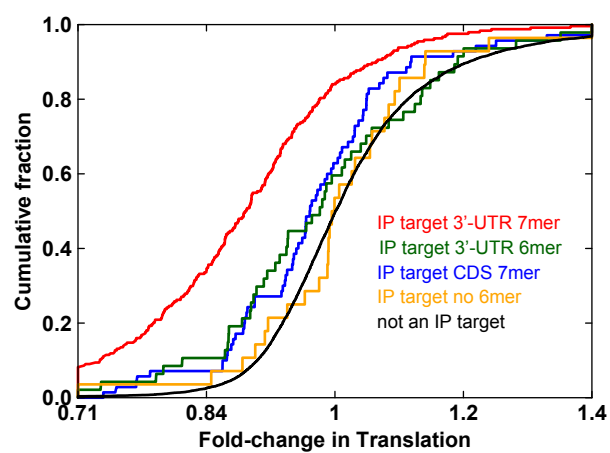

C

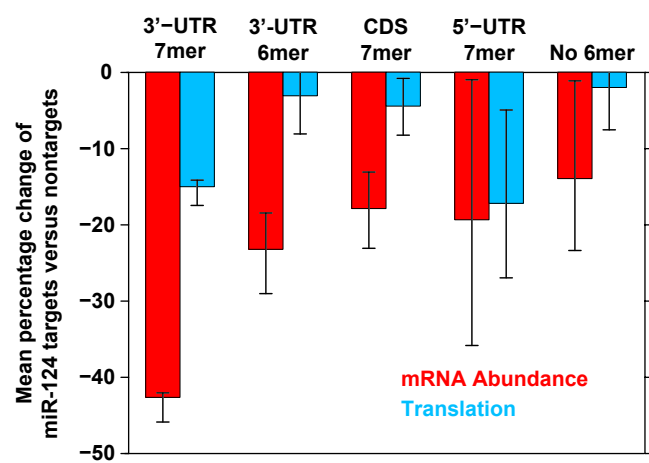

Supplement: Figure S9 — Changes in abundance and translation of miR-124 Ago IP targets with seed matches in 3′-UTRs, coding sequences and 5′-UTRs. (A) Cumulative distribution of the change in mRNA levels following transfection with miR-124 compared to mock. This analysis compares miR-124 Ago IP targets (1% local FDR) with at least one 3′-UTR 7mer seed match, but no coding sequence or 5′-UTR 7mer seed matches (red, 244), IP targets with at least one 3′-UTR 6mer seed match (green, 47), but no 3′-UTR, coding sequence, or 5′-UTR 7mer seed matches, IP targets with at least one coding sequence 7mer seed match, but no 3′-UTR or 5′-UTR 7mer seed matches (blue, 70), IP targets that lacked a 6mer seed match in the 3′-UTR, coding sequence, or 5′-UTR (orange,23), and nontargets (7385, black). This analysis compares Ago IP targets (red) versus nontargets (black). (B) Cumulative distribution of the change in translation following transfection with miR-124 compared to mock. This analysis compares miR-124 Ago IP targets (1% local FDR) with at least one 3′-UTR 7mer seed match, but no coding sequence or 5′-UTR 7mer seed matches (red), IP targets with at least one 3′-UTR 6mer seed match (green), but no 7mer seed matches in the 3′-UTR, coding sequence, or 5′-UTR, IP targets with at least one coding sequence 7mer seed match, but no 7mer seed match in the 3′-UTR or 5′-UTR (blue), IP targets that lacked a 6mer seed match in the 3′-UTR, coding sequence, or 5′-UTR (orange), and nontargets (black). This analysis compares Ago IP targets (red) versus nontargets (black). (C) Bar plot of the average change in mRNA abundance (blue) and translation rate (red) of miR-124 Ago IP targets following transfection with miR-124. The average change in mRNA abundance and translation of targets was calculated by subtracting the average change of nontargets for the mRNA abundance and translation rate measurements following transfection with miR-124. This analysis compares miR-124 Ago IP targets (1% local FDR) with at least one 3′-UT [file pbio.1000238.s014.pdf]

A

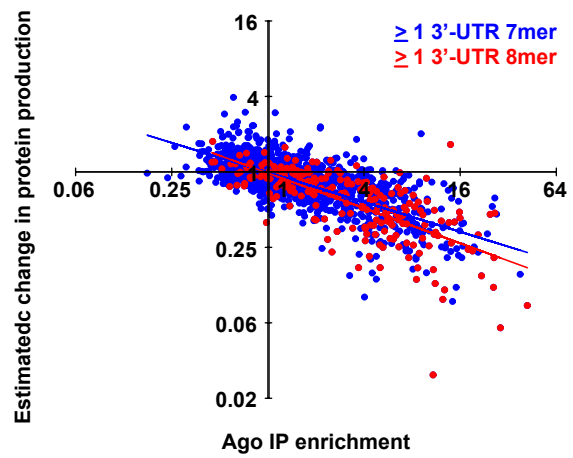

B

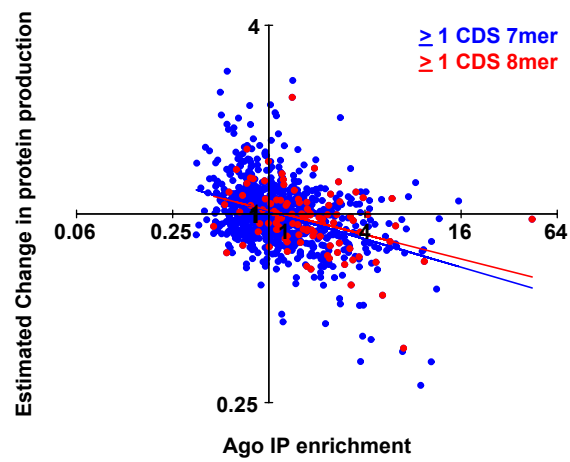

C

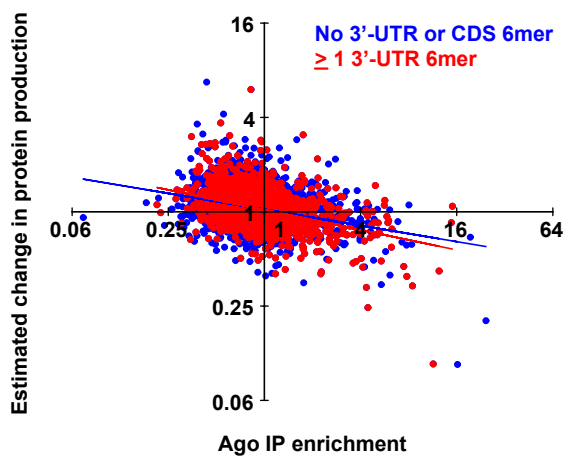

Supplement: Figure S10 — Efficiency of recruitment to Argonautes by miR-124 seed matches correlates with effects on both mRNA abundance and translation. (A) Scatterplot between changes in Ago IP enrichment (x-axis) following transfection with miR-124 compared to mock and estimated changes in protein production (Equation 2) (y-axis) for mRNAs with either 8mer seed matches (red dots) or 7mer seed matches (blue dots) to miR-124 in their 3′-UTRs. For 8mer seed matches, the slope of the least-squares fit of the log2 data is −0.46 (in linear space, −0.03), and the log2 Pearson correlation is −0.72. For 7mer seed matches, the slope of the least-squares fit of the log2 data is −0.39 (in linear space, −0.05), and the log2 Pearson correlation is −0.72. (B) Same as (A) except for mRNAs with seed matches to miR-124 in their coding sequences and no 7mer seed matches in their 3′-UTRs. For 8mer seed matches, the slope of the least-squares fit of the log2 data is −0.13 (in linear space, −0.008), and the log2 Pearson correlation is −0.39. For 7mer seed matches, the slope of the least-squares fit of the log2 data = −0.14 (in linear space, −0.02), and the log2 Pearson correlation is 0.38. (C) Same as in (A) except for mRNAs with 6mer seed matches to miR-124 in their 3′-UTR, but no 7mer seed match in their 3′-UTR or coding sequence (red), and mRNAs that lack 6mer seed matches in their 3′-UTR or coding sequence (blue). For 6mer seed matches, the slope of the least-squares fit of the log2 data is −0.21 (in linear space, −0.09), and the log2 Pearson correlation is −0.40. For mRNAs without 6mer seed matches, the slope of the least-squares fit of the log2 data is −0.13 (in linear space, −0.07), and the log2 Pearson correlation is −0.22. (1.71 MB PDF) [file pbio.1000238.s015.pdf]
