# Supplementary material for: Concordant Regulation of Translation and mRNA Abundance for Hundreds of Targets of a Human microRNA
Source: PLoS Biol. 2009 Nov 10;7(11):e1000238. doi: 10.1371/journal.pbio.1000238 (PMC2766070; doi:10.1371/journal.pbio.1000238)
Supplement: Figure S2 — Streptavidin-coated Dynal beads weakly enrich miR-124 targets after miR-124 transfection. (A) Supervised hierarchical clustering of the enrichment profiles of the 500 most enriched mRNAs in negative-control IPs from miR-124-transfected cells (blue) compared to mock-transfected cells (black). Rows correspond to mRNAs, and columns represent individual experiments. (B) Enrichment of seed matches to miR-124 in the 3′-UTRs of mRNAs nonspecifically associated with magnetic beads. The significance of enrichment of seed matches in Ago IP targets was measured with the hypergeometric distribution. (0.31 MB PDF) [file pbio.1000238.s007.pdf]

A

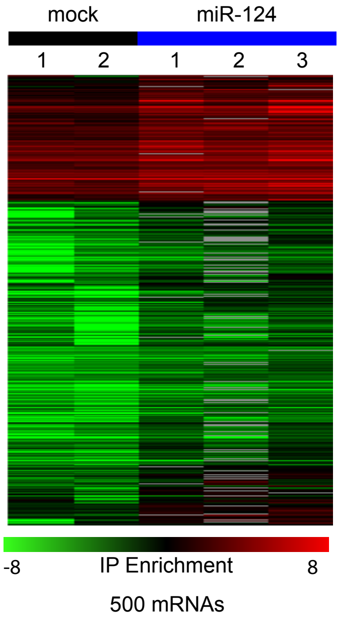

B

miR-124 negative control IP targets (500)

seed match	% of targets with match in 3'-UTR	$-\log_{10}(P)$	Sequence
8mer	7.5	9	GUGCCUUA
7mer_m8	22	11	GUGCCUUx
7mer_A1	16	3	xUGCCUUA
6mer_2-7	47	3	xUGCCUUx
6mer_3-8	40	6	GUGCCUxx

miR-124: 3'-ACCGUAAGUGGCGCACGGAAU-5'
