# Supplementary material for: Concordant Regulation of Translation and mRNA Abundance for Hundreds of Targets of a Human microRNA
Source: PLoS Biol. 2009 Nov 10;7(11):e1000238. doi: 10.1371/journal.pbio.1000238 (PMC2766070; doi:10.1371/journal.pbio.1000238)
Supplement: Figure S6 — Relationship between ribosome occupancy in mock-transfected cells and change in ribosome occupancy following transfection of miR-124. (A) Scatterplot between changes in ribosome occupancy (x-axis) and ribosome density (y-axis) for miR-124 Ago IP targets following transfection with miR-124 compared to mock. The gray line is a least-squares linear regression fit of the data (Spearman rank correlation = 0.45), and the red line is a moving average plot (window of 10). (B) The logarithm of the ratio of the average ribosome occupancy in miR-124-transfected cells to that in mock-transfected cells as a function of the average ribosome occupancy in mock-transfected cells (Spearman rank correlation = −0.78). Black circles correspond to mRNAs that were not enriched by the Ago IP following miR-124 transfection. Red circles correspond to mRNAs that were enriched by the Ago IP following miR-124 transfection (1% local FDR). The green curve represents a Lowess smoothed fit of the data. The gray curve shows the maximum possible increase in ribosome occupancy in miR-124 cells compared to mock cells. (C) The ratio in the average ribosome occupancy in miR-124-transfected cells versus mock-transfected cells minus the Lowess fit of the data (green points in (B)) as a function of the average ribosome occupancy in mock-transfected cells. (D) Scatterplot of changes in mRNA abundance (x-axis) versus changes in translation rate (y-axis) for Ago IP targets following transfection with miR-124. The slope of the least-squares fit of the data is 0.24 (in linear space, 0.36), and the Pearson correlation is 0.60. This is Figure 7 replotted to allow side-by-side comparison with (E). (E) Same as in (D), except that the changes in translation rate were obtained using the smoothed-fit adjusted ribosome occupancy measurements (C). The slope of the least-squares fit of the data is 0.20 (in linear space, 0.29), and the Pearson correlation is 0.59. (3.06 MB PDF) [file pbio.1000238.s011.pdf]

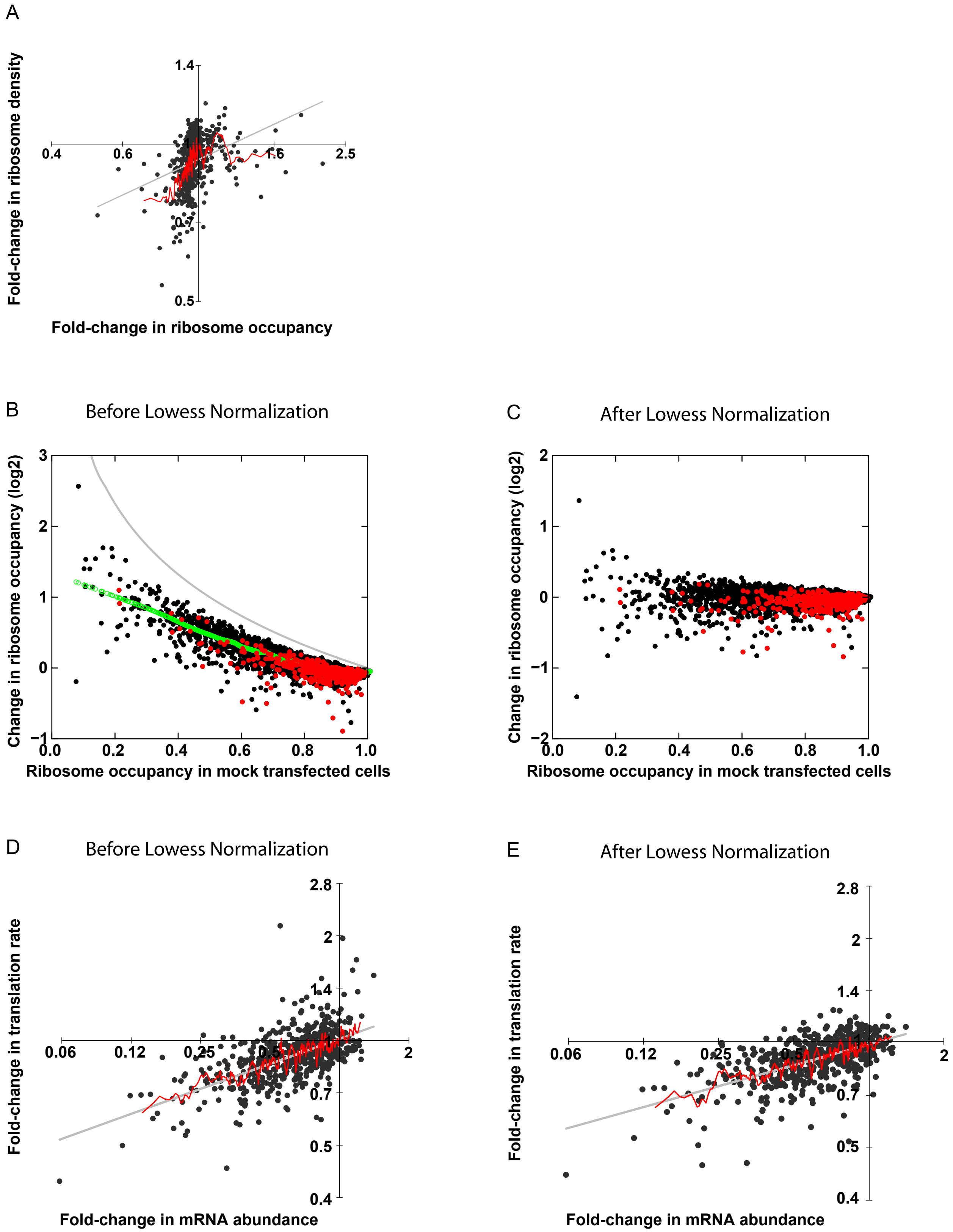

A
Fold-change in ribosome density
1.4
0.7
0.5
0.4
0.6
1
1.6
2.5
Fold-change in ribosome occupancy
B
Before Lowess Normalization
Change in ribosome occupancy (log2)
3
2
1
0
−1
0.0
0.2
0.4
0.6
0.8
1.0
Ribosome occupancy in mock transfected cells
C
After Lowess Normalization
Change in ribosome occupancy (log2)
2
1
0
−1
−2
0.0
0.2
0.4
0.6
0.8
1.0
Ribosome occupancy in mock transfected cells
D
Before Lowess Normalization
Fold-change in translation rate
2.8
2
1.4
0.7
0.5
0.4
0.06
0.12
0.25
0.5
2
Fold-change in mRNA abundance
E
After Lowess Normalization
Fold-change in translation rate
2.8
2
1.4
0.7
0.5
0.4
0.06
0.12
0.25
0.5
1
2
Fold-change in mRNA abundance
