# Supplementary material for: Concordant Regulation of Translation and mRNA Abundance for Hundreds of Targets of a Human microRNA
Source: PLoS Biol. 2009 Nov 10;7(11):e1000238. doi: 10.1371/journal.pbio.1000238 (PMC2766070; doi:10.1371/journal.pbio.1000238)
Supplement: Table S1 — Summary of miR-124 targets for Western blot analysis. (0.02 MB PDF) [file pbio.1000238.s016.pdf]

name	Specific Band	Estimated Change	Observed Change	IP Rank	3UTR 6mer-2_7	3UTR 6mer-3_8	3UTR 7mer-m8	3UTR 7mer-A1	3UTR 8mer	CDS 6mer-2_7	CDS 6mer-3_8	CDS 7mer-m8	CDS 7mer-A1	CDS 8mer
ITGB1	-	0.21	N/A	16	1	1	1	1	1	1	0	0	0	1
RNF128	+	0.27	0.92	50	1	1	1	1	1	0	2	0	0	0
MAPK14	+	0.31	0.28	64	1	2	1	1	1	0	0	0	0	0
CDK4	+	0.40	0.49	391	2	0	0	1	0	1	1	0	0	0
NRAS	+	0.44	0.51	138	1	1	1	0	0	0	0	0	0	0
AHR	+	0.48	0.36	26	1	2	1	1	1	4	1	1	1	0
DUSP9	+	0.49	0.55	467	0	1	0	0	0	0	2	0	0	0
DHCR24	+	0.56	0.38	164	1	1	1	1	1	0	3	0	0	0
PTPN11	+	0.64	0.66	644	3	2	1	0	0	0	0	0	0	0
ACTN4	+	0.77	0.86	553	1	1	1	0	0	3	0	0	0	0
AURKA	+	0.83	0.86	322	1	1	1	1	1	0	0	0	0	0
PLK1	+	1.04	0.91	297	0	0	0	0	0	0	1	0	0	0
TUBA1A	+	1.12	0.99	437	0	0	0	0	0	2	3	2	0	0
PTBP2	-	1.63	N/A	69	1	1	0	0	1	3	2	0	0	3
