# Supplementary material for: Concordant Regulation of Translation and mRNA Abundance for Hundreds of Targets of a Human microRNA
Source: PLoS Biol. 2009 Nov 10;7(11):e1000238. doi: 10.1371/journal.pbio.1000238 (PMC2766070; doi:10.1371/journal.pbio.1000238)
Supplement: Text S1 — miRNA-effector complexes appear to nonspecifically bind streptavidin-coated Dynal beads. (0.03 MB DOC) [file pbio.1000238.s018.doc]

**Text S1 miRNA-effector complexes appear to nonspecifically bind streptavidin coated Dynal beads.**

One explanation for the high correlation (r=0.6) we observed between the enrichment profiles of the Ago IPs and negative controls is that the “background” signature of the negative control IPs was in part driven by non-specific binding of miRNA effector proteins, such as the Agos, to the beads in the absence of an Ago-specific antibody. We reasoned that if the beads were nonspecifically enriching miRNA effector proteins, there would be enrichment for mRNAs that are recruited to Agos by miR-124 in miR-124 negative control IPs compared to negative control IPs from mock transfected cells.

To test this hypothesis, we determined if mRNAs most enriched in miR-124 negative control IPs compared to negative control IPs from mock transfected cells were more likely to contain seed matches to miR-124 in their 3’UTRs than expected by chance. First, we tested if any RNAs were specifically recruited to the beads by miR-124 using SAM to compare negative control IPs from mock versus miR-124 transfected cells. Only two of the three samples from mock transfected cells yielded microarray data of high enough quality for use in this analysis. No mRNAs were significantly enriched in the miR-124 negative control IPs at a 1%, 10%, or 50% FDR. However, hierarchical cluster analysis of the 500 most enriched sequences from SAM analysis (without recourse to statistical significance) segregated the miR-124 negative control IPs from the control IPs with mock transfected cells (Figure S1A). This population was slightly enriched for mRNAs that contained miR-124 seed matches in their 3’-UTRs (Figure S1B). These results suggest that the enrichment profiles from the negative control IPs are in part, generated from binding of miRNA effector proteins to the beads alone. The nonspecific binding is relatively weak as evidenced by the low amount of RNA and protein isolated from the beads (10x less than Ago IPs) and the fact that no individual mRNAs were enriched with high-statistical confidence in miR-124 negative control IPs compared to negative control IPs from mock transfected cells. Perhaps recruitment of miRNA effector proteins to the beads is driven by the association of Ago proteins with chaperones, such as Hsp90 [1,2] (unpublished data).

References

1. Landthaler M, Yalcin A, Tuschl T (2004) The human DiGeorge syndrome critical region gene 8 and Its D. melanogaster homolog are required for miRNA biogenesis. Curr Biol 14: 2162-2167.

2. Meister G, Landthaler M, Peters L, Chen PY, Urlaub H, et al. (2005) Identification of novel argonaute-associated proteins. Curr Biol 15: 2149-2155.
