# Supplementary material for: Concordant Regulation of Translation and mRNA Abundance for Hundreds of Targets of a Human microRNA
Source: PLoS Biol. 2009 Nov 10;7(11):e1000238. doi: 10.1371/journal.pbio.1000238 (PMC2766070; doi:10.1371/journal.pbio.1000238)
Supplement: Text S2 — Enrichment of seed matches to highly expressed miRNAs in Ago IPs from mock-transfected cells. (0.03 MB DOC) [file pbio.1000238.s019.doc]

**Text S2 Enrichment of Seed Matches to Highly-Expressed miRNAs in Ago IPs from Mock Transfected Cells.**

To test whether association with Ago is largely a reflection of the relative occupancy of each mRNA with the suite of endogenously expressed miRNAs in HEK293T cells, we first determined if there were sequence motifs in mRNA untranslated regions that significantly correlated with Ago IP enrichment, and if so, if these motifs corresponded to known miRNA seed matches. Such sequence motifs might be identifiable if one or a few highly expressed miRNAs dominated the IP enrichment. To search for possible sequence motifs, we used the motif prediction algorithm FIRE[1]. Two motifs, located in 3’-UTRs, significantly correlated with Ago IP enrichment; these motifs overlapped and corresponded to seed match sequences of the very abundant miR-17-5p/20/92/106/591.d and miR-19 families of miRNAs. mRNAs with seed matches to these miRNAs in good sequence contexts (TargetScan 4.2, context score < -0.3) were more likely to be enriched in Ago IPs than mRNAs with seed matches in poor sequence contexts (context score > -0.1) (for miR-17: 2.9 mean fold-enrichment for mRNAs in good context versus 0.91 mean fold-enrichment for mRNAs in poor context, p < 10^-12^, one-sided Kolmogorov-Smirnov test) [2]. This result suggests that these abundant miRNAs contribute significantly to recruitment to RISC and Ago IP enrichment. For a majority of expressed miRNAs, however, we did not find that predicted targets with seed matches in a good context were more likely to be enriched than mRNAs with seed matches in a poor context. This negative finding may result because mRNA enrichment in Ago IPs is mediated by multiple miRNAs so that the enrichment signal for any specific miRNA is diluted.

References

1. Elemento O, Slonim N, Tavazoie S (2007) A universal framework for regulatory element discovery across all genomes and data types. Mol Cell 28: 337-350.

2. Grimson A, Farh KK, Johnston WK, Garrett-Engele P, Lim LP, et al. (2007) MicroRNA targeting specificity in mammals: determinants beyond seed pairing. Mol Cell 27: 91-105.
