# Supplementary material for: Concordant Regulation of Translation and mRNA Abundance for Hundreds of Targets of a Human microRNA
Source: PLoS Biol. 2009 Nov 10;7(11):e1000238. doi: 10.1371/journal.pbio.1000238 (PMC2766070; doi:10.1371/journal.pbio.1000238)
Supplement: Text S3 — Relationship between ribosome occupancy in mock-transfected cells and changes in ribosome occupancy following transfection of miR-124. (0.03 MB DOC) [file pbio.1000238.s020.doc]

**Text S3 Relationship Between Ribosome Occupancy in Mock-Transfected Cells and the Change in Ribosome Occupancy Following Transfection of miR-124.**

Twenty-one miR-124 Ago IP targets appeared to increase at least 20% in ribosome occupancy due to the presence of miR-124. Strikingly, a large fraction of mRNAs that increased at least 20% in ribosome occupancy encode transcription factors (11/21, *p* < 10^-4^).

We asked if miR-124 targets that increased at least 20% in ribosome occupancy behave anomalously in other ways, which might provide some insight into their apparent increase in occupancy. We compared mRNAs that increased at least 20% in ribosome occupancy to the other miR-124 Ago IP targets with regards to several factors. While there was no difference in terms of the fraction of each group that contained seed matches to miR-124 or their ribosome density, targets that increased in ribosome occupancy were less likely to decrease in abundance (13% vs 33%) and had much lower ribosome occupancy in untreated cells than the other miR-124 targets (49% vs 87%).

The fact that the mRNA targets that appeared to increase in ribosome occupancy tended to have low occupancy in mock transfected cells prompted us to ask if this was a general phenomenon. We found that across all mRNAs there was a strong negative correlation between the miR-124-induced change in ribosome occupancy of a gene’s mRNAs and its ribosome occupancy in mock transfected cells (Spearman r = -0.78) (Figure S6B). This relationship could, in principle, be due to errors in our microarray measurements, which would tend to have a larger effect on the estimated ribosome occupancy as ribosome occupancy decreases. If this were the case we would expect significant correlations between the change in ribosome occupancy and ribosome occupancy between biological replicate experiments. However, the relationship between the change in ribosome occupancy as a function of ribosome occupancy is much weaker between biological replicates (r = -0.07, 0.24 and 0.02 for mock replicates and -0.11 for miR-124 replicate) than between mock and miR-124 experiments (r = -0.55, -0.45, -0.66, -0.60, -0.74 and -0.77 between mock and miR-124 experiments), which suggests it may actually be due to bona fide biological differences between mock and miR-124 transfected cells. Indeed, mRNAs that have a low initial ribosome occupancy have more potential to increase in ribosome occupancy between experimental conditions (Figure S6B, gray curve). For instance, a gene whose mRNAs are 10% occupied can increase up to 10-fold, while a gene whose mRNAs are 90% occupied increase no more than 1.1 fold.

While the origin of the relationship between changes in ribosome occupancy and starting ribosome occupancy might be biological, we still wished to investigate the effect of this potential artifact on our results. We attempted to remove the relationship between changes in ribosome occupancy and starting ribosome occupancy by fitting a locally weighted scatterplot smoothing (Lowess) function to the scatterplot between mock ribosome occupancy and change in ribosome occupancy and subtracting the fitted values (Figure S6B – green circles) [1]. As expected, after this transformation, there was no longer a correlation between ribosome occupancy in mock experiments and the miR-124-induced change in ribosome occupancy (Figure S6C). The overall effects on ribosome occupancy and translation were very similar between the renormalized data and raw data (mean difference in occupancy between targets and nontargets with Lowess normalization = 4%, and without Lowess normalization = 4%) and the correlation between changes in translation and abundance for miR-124 Ago IP targets were very similar (Lowess-normalized = 0.59, without Lowess normalization = 0.60) (Figure S6D and S6E). However, the group of mRNAs that, prior to this transformation, had appeared to increase in translation in the miR-124 transfected cells no longer appeared to do so after the Lowess normalization. Following the Lowess normalization, the IP targets that previously appeared to increase in occupancy by at least 20% had, on average, no change in occupancy, there were no IP targets with > 13% apparent increase in occupancy, and there was no functional bias among mRNAs that appeared to increase in occupancy.

References

1. Cleveland WS (1979) Robust Locally Weighted Fitting and Smoothing Scatterplots. Journal of the American Statistical Association 74: 829-836.
