# Supplementary material for: Concordant Regulation of Translation and mRNA Abundance for Hundreds of Targets of a Human microRNA
Source: PLoS Biol. 2009 Nov 10;7(11):e1000238. doi: 10.1371/journal.pbio.1000238 (PMC2766070; doi:10.1371/journal.pbio.1000238)
Supplement: Text S4 — Evaluation of the significance of the correlation between changes in mRNA abundance and translation of miR-124 Ago IP targets following transfection with miR-124. (0.05 MB DOC) [file pbio.1000238.s021.doc]

**Text S4. Evaluation of the Significance of the Correlation between Changes in mRNA Abundance and Translation of miR-124 Ago IP Targets Following Transfection with miR-124.**

We evaluated the significance of the correlation between mRNA abundance changes and estimated translation rate changes in several ways. First, we asked from a purely statistical perspective, what was the likelihood of observing the correlation by chance, given the values we had. To test this, we calculated the correlation between expression and translation after resampling the expression data. We did this 100,000 times, fit the data to a normal distribution, and estimated the likelihood of observing the correlation by chance. Using this methodology, we found the likelihood of getting a correlation of 0.60 by chance is 10^-45^ (Figure S7A). Second, we tested if the correlation persisted in nontarget mRNAs. We found that the Pearson correlation between expression changes and translation changes for mRNAs that are not IP targets was 0.11. We ranked mRNAs by IP enrichment and plotted the correlation between changes in expression and translation as a moving window, and found that the correlation monotonically falls off to baseline level after the several hundred most enriched mRNAs, indicating the correlation is specific to mRNAs most enriched in Ago IPs due to the presence of miR-124 (Figure S7B). Third, we tested if the correlation was specific to miR-124 targets, or if the observed relationship between mRNA abundance and translation was a general phenomenon. It is well documented that changes in abundance and translation tend to correlate and so it may not be specific to miR-124 response, but rather reflect a common relationship between effects on translation and mRNA abundance in this system [1-13]. To estimate the difference in the strength of the correlation between translation and mRNA abundance we chose nontarget mRNAs that decreased at the mRNA level similarly to miR-124 target mRNAs. We had to focus on miR-124 targets that changed less than 40% in mRNA abundance as there were not any nontargets that decreased more than 40%. We compared the miR-124 IP targets that decreased less than 40% in mRNA abundance to 10,000 random sets of nontargets of the same size with similar distributions of expression changes (t-test, p > 0.001). The average correlation between changes in mRNA abundance and translation rate for the 10,000 sets of nontargets was 0.14, whereas the correlation for miR-124 IP targets changing in mRNA abundance < 40% was 0.30 (1/10,000 permuted nontargets sets had a correlation greater than the IP targets – we estimated the actual p-value using normal distribution function to be < 10^-5^) (Figure S7C). We also observed a modest, but significant, positive correlation between changes in expression and translation for mRNAs whose abundance increased (0.19 for 662 mRNAs that increased at least 25%.) These data suggest changes in abundance and translation rate are generally correlated under these growth conditions, but tend to be more so for miR-124 targets.

References

1. Bandyopadhyay R, Coutts M, Krowczynska A, Brawerman G (1990) Nuclease activity associated with mammalian mRNA in its native state: possible basis for selectivity in mRNA decay. Mol Cell Biol 10: 2060-2069.

2. Caruccio N, Ross J (1994) Purification of a human polyribosome-associated 3' to 5' exoribonuclease. J Biol Chem 269: 31814-31821.

3. Grafi G, Sela I, Galili G (1993) Translational regulation of human beta interferon mRNA: association of the 3' AU-rich sequence with the poly(A) tail reduces translation efficiency in vitro. Mol Cell Biol 13: 3487-3493.

4. Jacobson A, Peltz SW (1996) Interrelationships of the pathways of mRNA decay and translation in eukaryotic cells. Annu Rev Biochem 65: 693-739.

5. Kruys V, Marinx O, Shaw G, Deschamps J, Huez G (1989) Translational blockade imposed by cytokine-derived UA-rich sequences. Science 245: 852-855.

6. Kruys V, Wathelet M, Poupart P, Contreras R, Fiers W, et al. (1987) The 3' untranslated region of the human interferon-beta mRNA has an inhibitory effect on translation. Proc Natl Acad Sci U S A 84: 6030-6034.

7. Kruys VI, Wathelet MG, Huez GA (1988) Identification of a translation inhibitory element (TIE) in the 3' untranslated region of the human interferon-beta mRNA. Gene 72: 191-200.

8. Marinx O, Bertrand S, Karsenti E, Huez G, Kruys V (1994) Fertilization of Xenopus eggs imposes a complete translational arrest of mRNAs containing 3'UUAUUUAU elements. FEBS Lett 345: 107-112.

9. Muhlrad D, Decker CJ, Parker R (1995) Turnover mechanisms of the stable yeast PGK1 mRNA. Mol Cell Biol 15: 2145-2156.

10. Chu CY, Rana TM (2006) Translation repression in human cells by microRNA-induced gene silencing requires RCK/p54. PLoS Biol 4: e210.

11. Coller J, Parker R (2005) General translational repression by activators of mRNA decapping. Cell 122: 875-886.

12. Kawai T, Fan J, Mazan-Mamczarz K, Gorospe M (2004) Global mRNA stabilization preferentially linked to translational repression during the endoplasmic reticulum stress response. Mol Cell Biol 24: 6773-6787.

13. Sheth U, Parker R (2003) Decapping and decay of messenger RNA occur in cytoplasmic processing bodies. Science 300: 805-808.
